# A ligand-centered framework for γδ T cell activation in colorectal cancer revealed by single-cell and transformer-based perturbation

**DOI:** 10.3389/fimmu.2025.1715827

**Published:** 2026-01-13

**Authors:** Ran Ran, Douglas K. Brubaker

**Affiliations:** 1 Center for Global Health and Diseases, Department of Pathology, Case Western Reserve University, Cleveland, OH, United States; 2 The Blood, Heart, Lung, and Immunology Research Center, Case Western Reserve University, University Hospitals of Cleveland, Cleveland, OH, United States

**Keywords:** cancer colorectal, deep learn ing, gamma delta (γδ) T cells, ligand, perturbation prediction, transformer

## Abstract

Understanding the activation mechanisms of γδ T cells in colorectal cancer (CRC) is critical for harnessing their therapeutic potential. Here, using an atlas of human CRC-infiltrating γδ T cells that we built by integrating multiple single-cell RNA-seq datasets, we developed a γδ T cell-refined ligand inference pipeline by combining differential gene expression, gene regulatory network prediction, ligand inference, and in silico perturbation analysis. This approach identified ligands, including IL-15 and TNFSF9 (4-1BBL), as candidates promoting γδ T cell effector function and highlighted NCR2 and KLRC3 (NKG2E), whose in silico overexpression was associated with γδ T cell activation. Ligand enrichment analyses further indicated that monocytes and dendritic cells are key contributors to γδ T cell activation in the tumor microenvironment. Our results also highlighted transcription factors IKZF1, FOSL2, and FOXO1 in the less activated γδ T cells and IRF1, KLF2, and BHLHE40 in the effector γδ T cells that plausibly regulated the differential activation state. Together, our results offer a systems-level view of the signaling and transcriptional programs governing γδ T cell phenotypes in CRC and provide a foundation for γδ T cell-based immunotherapies with enhanced antitumor functions.

## Introduction

Colorectal cancer (CRC) is one of the most frequently diagnosed cancers and the leading cause of cancer-related deaths (1–3). It originates in the colon or rectum and is characterized by the abnormal proliferation of epithelial cells (4). Surgery is the primary treatment for advanced resectable CRC, yet recurrence occurs in a substantial proportion of patients with stage II or III disease (5, 6). Most recurrences occur within 5 years after surgery, and the relapse significantly increases the risk of metastasis and decreases the survival rate (5, 7, 8). For non-resectable CRC, radiotherapy and chemotherapy are standard-of-care therapies that can reduce tumor burden (9, 10) but are limited by systemic toxicity, adverse effects, and a lack of tumor specificity (11). A substantial proportion of patients eventually develop incurable recurrent disease, even after standard treatments (11). Therefore, there is a need for novel treatments that can be used in addition to conventional treatments to prevent recurrence.

γδ T cells are a unique subset of immune cells with potent anti-tumoral ability (12, 13). They can be activated independently of classical MHC-mediated antigen presentation and, in some cases, through TCR-independent mechanisms involving the engagement of stress-induced ligands on target cells (14). Although γδ T cells account for less than 5% of human blood T lymphocytes, their proportion is significantly higher in the colon mucosa (15). They account for 10-40% of T cells in the epithelium monolayer (16–18), where CRC oncogenesis usually begins (19). These cells exhibit dynamic surveillance behavior, migrating through the intestinal epithelium in a flossing-like manner and traversing the basement membrane to access the lamina propria (15, 20). γδ T cells have been shown to inhibit intestinal tumor growth via CD103-E-cadherin interactions, highlighting their role in maintaining epithelial integrity and tumor surveillance under homeostatic conditions (21). Therefore, they are promising candidates for immunotherapeutic strategies because they are naturally present in the tumor microenvironment, exhibit strong tissue infiltration, and recognize targets in an MHC-independent manner, enabling allogeneic transfer.

However, the mechanism underlying the activation of γδ T cells in CRC remains unclear. In our previous study, we integrated single-cell RNA sequencing (scRNA-seq) data from multiple CRC studies and revealed diverse states among CRC-infiltrating γδ T cells. These include a substantial population of unexhausted, tissue-resident bystanders expressing *ITGAE* and *ITGA1*, as well as highly cytotoxic effector cells expressing *IFNG* (22). We therefore hypothesize that identifying the molecular cues driving the activation of these bystanders could support tumor cell elimination and reduce CRC recurrence, given that recurrence often arises from residual tumor cells (7).

It is well established that intraepithelial γδ T cells interact closely with colon epithelial cells under homeostatic conditions (21). Through both T cell receptor (TCR)-dependent and TCR-independent pathways, they detect ligands expressed on epithelial cells or present in the local microenvironment. T cell activation also depends on the co-stimulatory ligands they engage and the cytokines they receive. Taken together, these insights support the hypothesis that γδ T cells may undergo a shift from a less activated to a more activated state through TCR-independent ligand-receptor interactions.

Single-cell RNA sequencing is transforming CRC research by enabling high-resolution profiling of gene expression in heterogeneous tumor tissues, capturing the activity of individual cell types, including tumor-infiltrating immune cells. Here, we combine our previous multi-study integration of γδ T cell scRNA-seq data with gene regulatory network (GRN) inference and large language model (LLM)-based in silico gene perturbation to uncover the molecular drivers of γδ T cell activation in CRC. Through this integrative approach, we identified genes that correlate with, and may potentially drive, the activation transition, and made backward inferences about upstream ligands regulating them via signaling pathways.

## Result

### CRC-infiltrating γδ T subsets have varied activation levels

**Figure 1:**
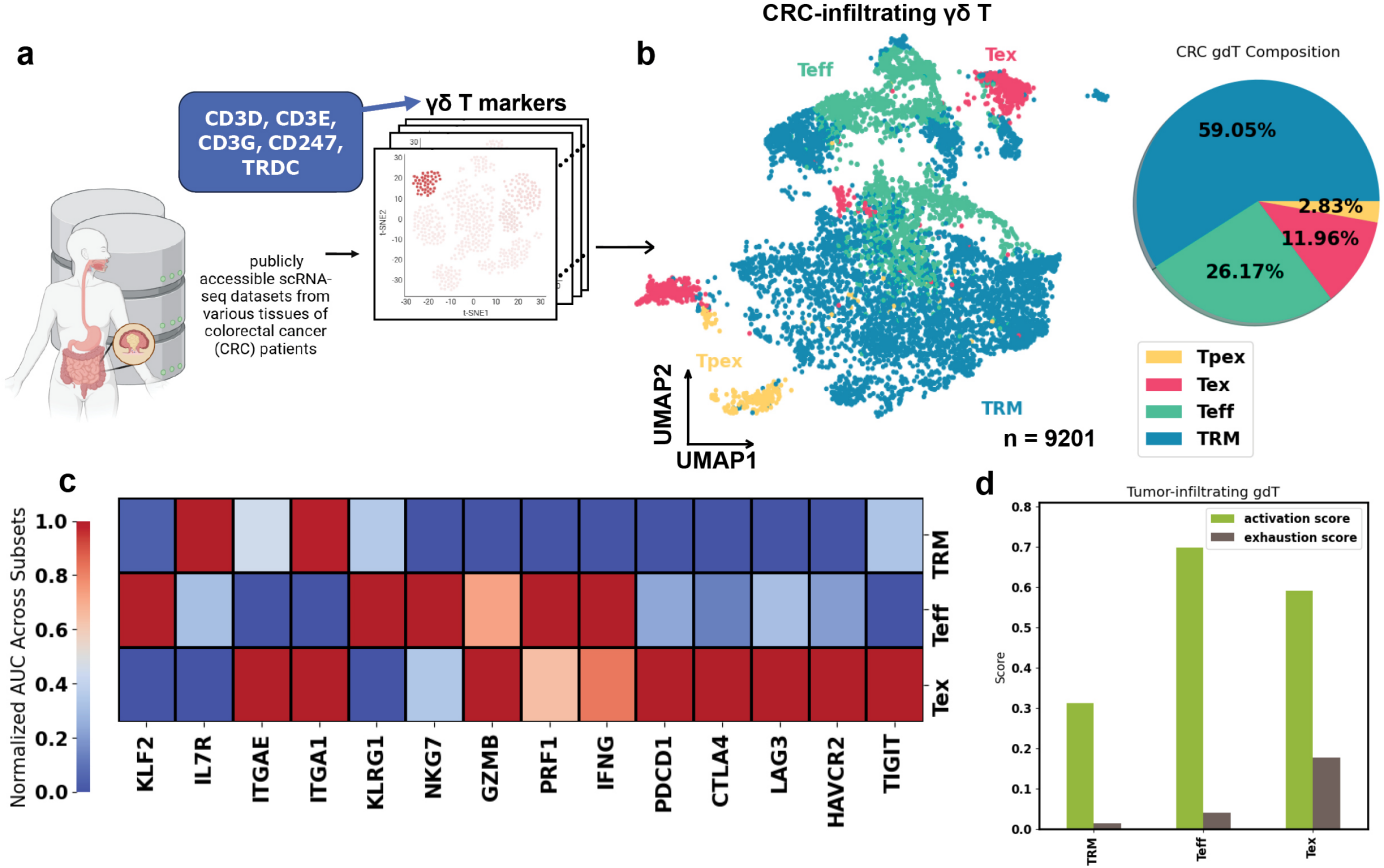
Overview of the heterogeneous human CRC γδ T cells. **(a)** Using the minimum set of γδ T cell transcriptomics markers we developed, we examined publicly available CRC scRNA-seq datasets and identified γδ T clusters. They are integrated to build a human CRC γδ T cells dataset. **(b)** (Left) Uniform manifold approximation and projection (UMAP) of γδ T cells showing subsets: Teff (effector-like T cells), TRM (tissue-resident memory T cells), Tpex (progenitor exhausted T cells), and Tex (exhausted T cells). (Right) Composition of γδ T cell subtypes in the tumor. **(c)** Heatmap showing the T cell function-related marker RNA expression in TRM, Teff, and Tex. Expression has been normalized across cell types. **(d)** Bar plot showing the effector activation score and exhaustion score in γδ T cell subsets.

In our previous study, we integrated γδ T cells from multiple human CRC datasets to construct a comprehensive cell atlas with over 18,000 γδ T cells, of which 9,201 were identified as isolated from tumors by the data providers, across diverse anatomical sites, patient demographics, and disease stages, providing a broad view of γδ T cell transcriptional states in CRC (22) (**Figure 1a**). We performed clustering on this integrated dataset and identified γδ T cell subtypes based on canonical αβ T cell functional subset marker genes curated from the literature, including effector T cells (Teff), tissue-resident memory T cells (TRM), progenitor exhausted T cells (Tpex), and exhausted T cells (Tex), all present in both tumor and adjacent normal tissues, with Teff and Tex more enriched in tumors. Notably, although we use the term “exhausted” to describe γδ T cells that express canonical exhaustion markers, emerging evidence indicates these cells may not be functionally impaired (23, 24). A more accurate descriptor is therefore “exhaustion-marker-expressing” γδ T cells. For brevity, however, we will continue to refer to this population as “Tex” throughout the manuscript. We observed that the TRM-like population, the major subtype among tumor-infiltrating γδ T cells in our integrated dataset (**Figure 1b**), did not highly express conventional exhaustion markers such as *PDCD1* and *TIGIT* or effector-related genes including *GZMB*, *NKG7*, *PRF1*, or *IFNG* (**Figure 1c**). Gene set scoring based on effector genes (*IL2RA*, *CD38*, *NKG7*, *GZMB*, *PRF1*, *IFNG*) and exhaustion genes (*PDCD1*, *CTLA4*, *LAG3*, *HAVCR2*, *TIGIT*) further confirmed that the TRM-like population exhibited the lowest scores for both effector and exhaustion signatures (**Figure 1d**).

**Figure 2:**
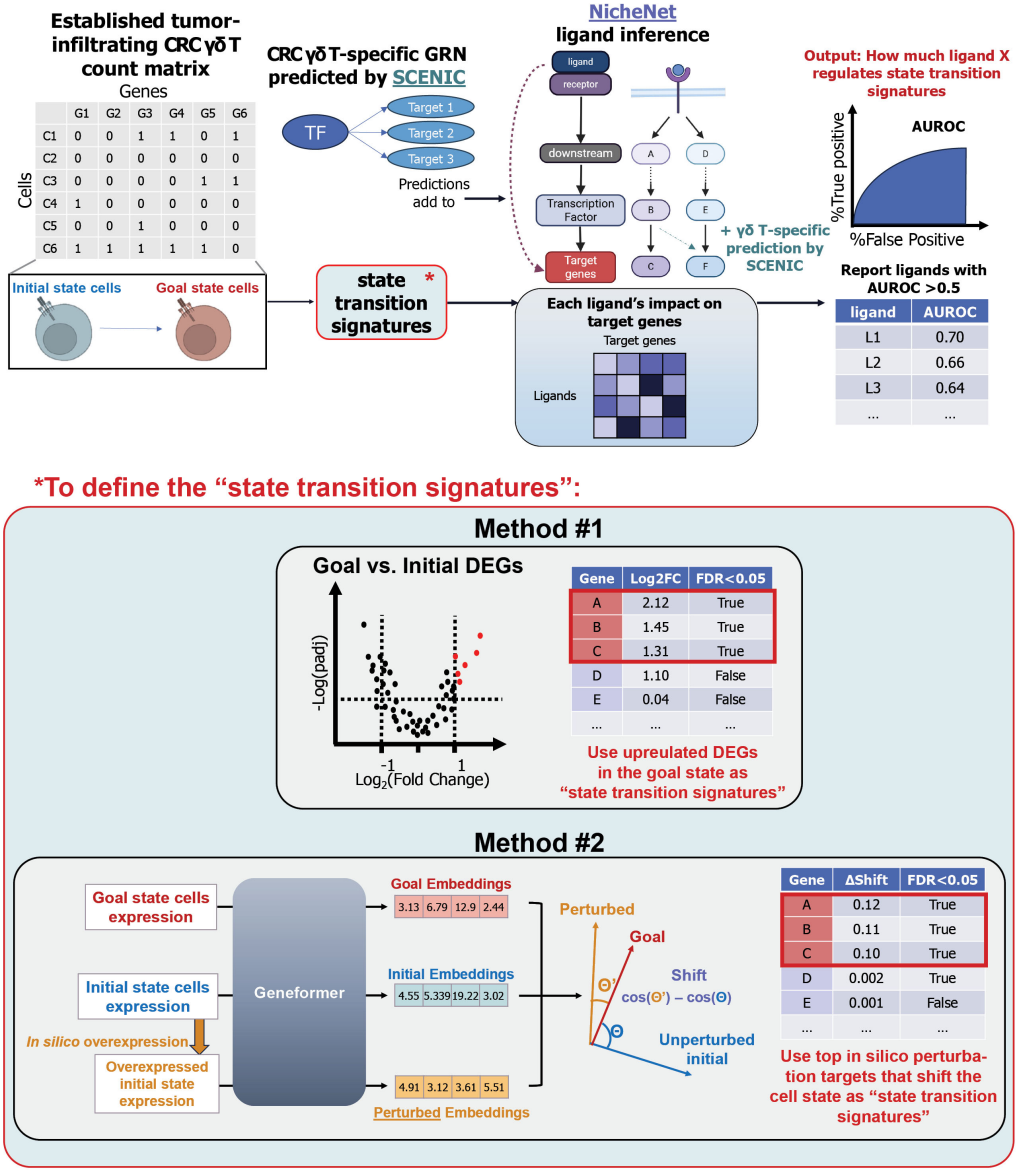
γδ T cell-refined ligand inference pipeline. Overview of the computational pipeline. We first define a potential cell state transition from an initial state (i.e., Teff) to a goal state (i.e., TRM) in the scRNA-seq count matrix. Then, we use NicheNet, a ligand inference tool that uses Personalized Page Rank on the constructed ligand to target gene network, known networks summarized by NicheNet authors from databases, and γδ T-specific networks predicted by SCENIC based on the co-expression of known TF with potential target genes in our data, to approximate the propagation of the signal in order to compute each ligand’s regulatory power on its target gene transcription. By computing the AUROC between the transition-associated signatures and each ligand to target genes’ regulatory potential vector, we can infer which ligand has the highest potential of regulating the given signatures. The transition signatures are calculated in two ways: differential gene expression analysis and in silico perturbation analysis using Geneformer, a language model. For visualization, the embedding is drawn as a 4-element array here as a schematic representation of a 512-dimensional vector.

Given the plasticity of T cells, we aimed to identify candidate ligands that could drive the potential transition from TRM-like bystanders to Teff cells in CRC. Using scRNA-seq data, we developed a pipeline that integrates multiple computational tools to perform ligand inference (**Figure 2**). At the core of this pipeline is NicheNet (25), a computational framework that performs a random walk on a ligand to target gene transcription network that is constructed from known ligand-receptor interactions, intracellular signaling, and gene regulatory networks to infer the ligands that are most influential in regulating a given set of genes. For the standard processing, we input differentially upregulated genes in the Teff compared to TRM as the signature associated with the state transition into NicheNet, which will output the ligands that most influence these differential expression events. We chose NicheNet primarily because it directly links ligands to downstream target gene transcription, rather than stopping at ligand-receptor co-expression. In addition, compared with other network-based approaches, NicheNet is explicitly receiver-centric, modeling how putative ligands affect the transcriptional programs of a specified receiver population. This receiver-focused design is particularly advantageous for rare cell types like CRC-infiltrating γδ T cells, where the identity of the dominant sender populations is not always obvious *a priori* in the complex TME. It also provides flexibility to interpret ligand sources and to prioritize potential experimental interventions on either ligands or receptors in follow-up work.

One challenge in this style of ligand inference is that the differentially expressed genes (DEGs) between TRM and Teff γδ T cells identify genes associated with their transcriptional differences, but such differences may reflect a mixture of upstream, downstream, and unrelated processes. To prioritize gene programs more likely to contribute to the transition between these states, we incorporated Geneformer (26), a large language model pre-trained on human scRNA-seq data for *in silico* perturbation analysis, into our pipeline to identify genes whose in silico overexpression is associated with a shift from a TRM-like to a Teff-like transcriptional profile. In our modified workflow, NicheNet was used to calculate another set of ligands that can regulate Geneformer-predicted gene expression events, leading to cell state transition. We chose Geneformer because it is pretrained exclusively on a large corpus of human single-cell data and provides a built-in perturbation function that directly links simulated changes in gene expression to shifts in the cell-state embedding, quantified using cosine similarity. Other large pretrained models such as scGPT (17) or scFoundation (18) can generate binned or continuous perturbed gene expression profiles, and in principle one could derive state embeddings and perform Geneformer-like quantification from those outputs. However, doing so would require customization of their original pipelines. In contrast, Geneformer offered a robust and immediately applicable framework for ranking ‘state-shifting’ genes in our TRM-to-Teff transition analysis, while we view alternative models as promising avenues for future extensions.

A further challenge in ligand signaling inference is that gene regulatory networks (GRN) are cell type-specific (27) and γδ T cells are a less well-studied population whose cellular programs may not be well-represented in prior knowledge networks. To address this limitation of existing workflows, we integrated ligand-receptor interactions relevant to γδ T cells, curated from the literature (14, 28–33), with SCENIC (34)-inferred transcription factor (TF)-target gene relationships into the NicheNet current default network. Using this γδ T cell-tailored network, we performed finely tuned γδ T cell-specific ligand inference to prioritize candidate ligands and gene expression programs associated with γδ T cell activation in CRC. We chose SCENIC for GRN inference primarily because it balances co-expression analysis with transcription factor motif enrichment, which is particularly advantageous when prior knowledge about a rare cell type like γδ T cells is limited. Unlike approaches that rely solely on enhancer-gene correlations [for example GRaNIE (12) or ANANSE (13)] or on sparse regression [for example Inferelator (14)], SCENIC combines intuitive tree-based gene-expression modeling with motif enrichment analysis around target gene promoters. This allows relatively robust identification of high-confidence TF-target interactions even in the absence of clear condition contrasts or additional omics layers such as ATAC-seq [required by methods like sc-compReg (15)] or lineage information [used by approaches such as scMTNI (16)]. This biologically interpretable framework, together with SCENIC’s broad adoption and mature software ecosystem, made it a practical choice for γδ T cell–specific GRN inference in our setting.

### Integration of SCENIC-predicted novel gene regulatory relationships maintains general predictive performance

**Figure 3:**
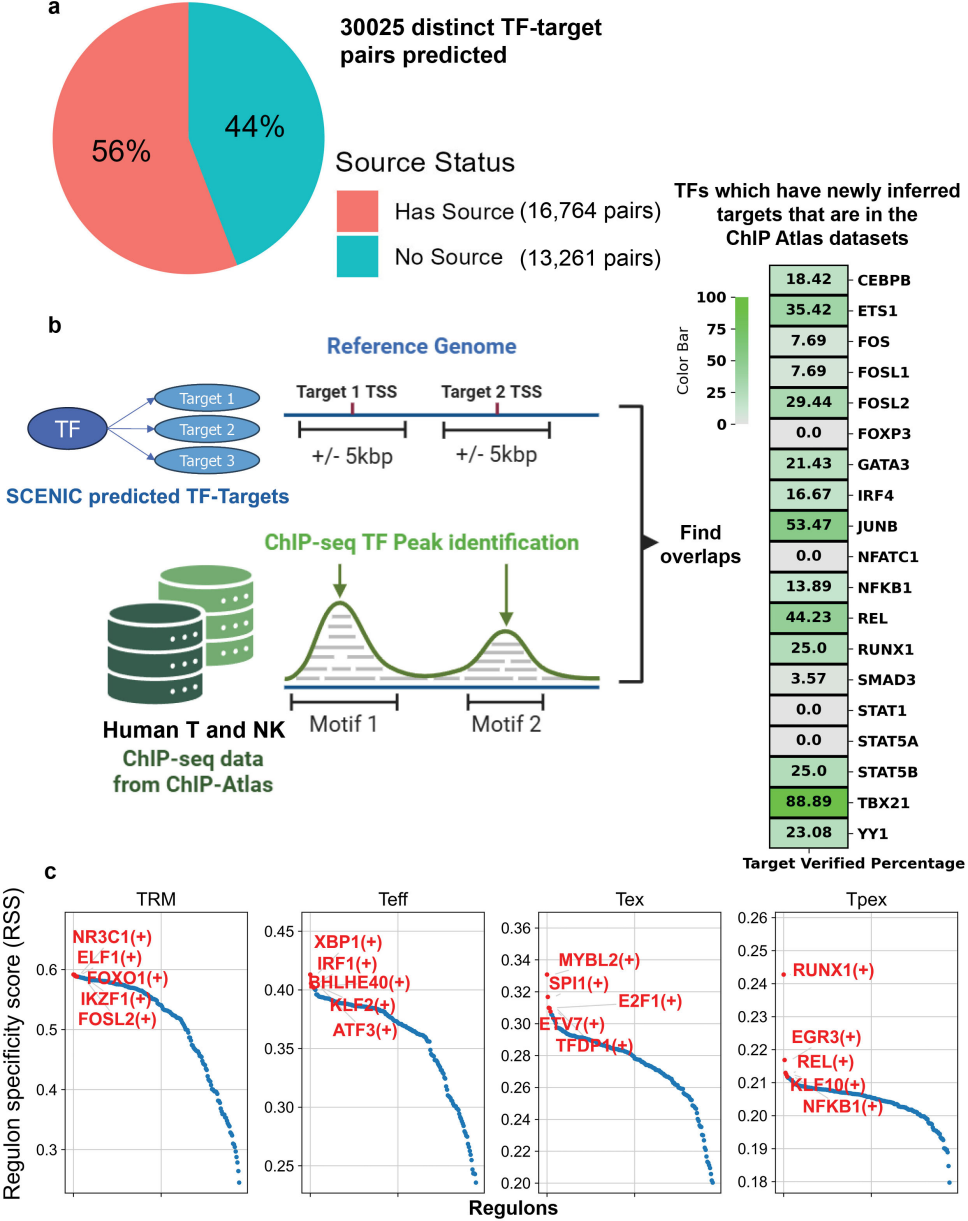
SCENIC predicts gene regulatory networks and TF activities in human CRC γδ T cells. **(a)** pie chart showing the percentage of SCENIC predicted TF-target gene pairs that are already in the NicheNet-summarized GRN. Has Source: the SCENIC-identified pair is in the NicheNet-summarized GRN version “21122021;” No source: the SCENIC-identified pair is not in the NicheNet-summarized GRN version “21122021.” **(b)** (Left) Workflow of using ChIP-seq data to validate SCENIC prediction that is not documented in NicheNet-summarized GRN version “21122021”. Overlap between a TF’s target genes TSS surroundings and that TF’s ChIP-seq peaks, if contained in the database, is calculated. (Right) Heatmap visualizing the percentage of TF’s SCENIC-predicted target genes can be found to overlap with a peak. **(c)** The regulon specificity score plot showing how the collective expression of a TF’s target genes in cells converges with the cell type labels, or in other words, how specific a TF’s regulatory activities are to the cell type. The x-axis is the ranked regulons.

The first step was to infer CRC-infiltrating γδ T cell-specific TF-target gene relationships from the integrated single-cell atlas. Using SCENIC, we identified 30,025 significant and distinct TF-target gene pairs in our γδ T cell atlas, of which 56% were already documented in the NicheNet default GRN (**Figure 3a**; full list of regulons in **Supplementary Table S1**). We used publicly available human T and NK cell ChIP-seq data to assess whether the remaining 44%, approximately 13,200 novel TF-target gene pairs specific to CRC-infiltrating γδ T cells, could be validated using an orthogonal experimental setup. From ChIP-Atlas (35–37), 19 of the predicted TFs had available peak information, and for 15 of them, ChIP-seq peaks were found within ±5 kb of the transcription start site (TSS) of at least a subset of their SCENIC-predicted target genes (**Figure 3b**). Regulon specificity scores across the four γδ T cell subsets indicated distinct TF regulatory programs, with TRM cells primarily regulated by TFs such as IKZF1 and FOXO1, Teff cells by KLF2 and interferon regulatory factor 1 (IRF1), Tex cells by MYBL2 and SPI1, and Tpex cells by RUNX1, EGR3, and NFKB1 (**Figure 3c**).

**Figure 4:**
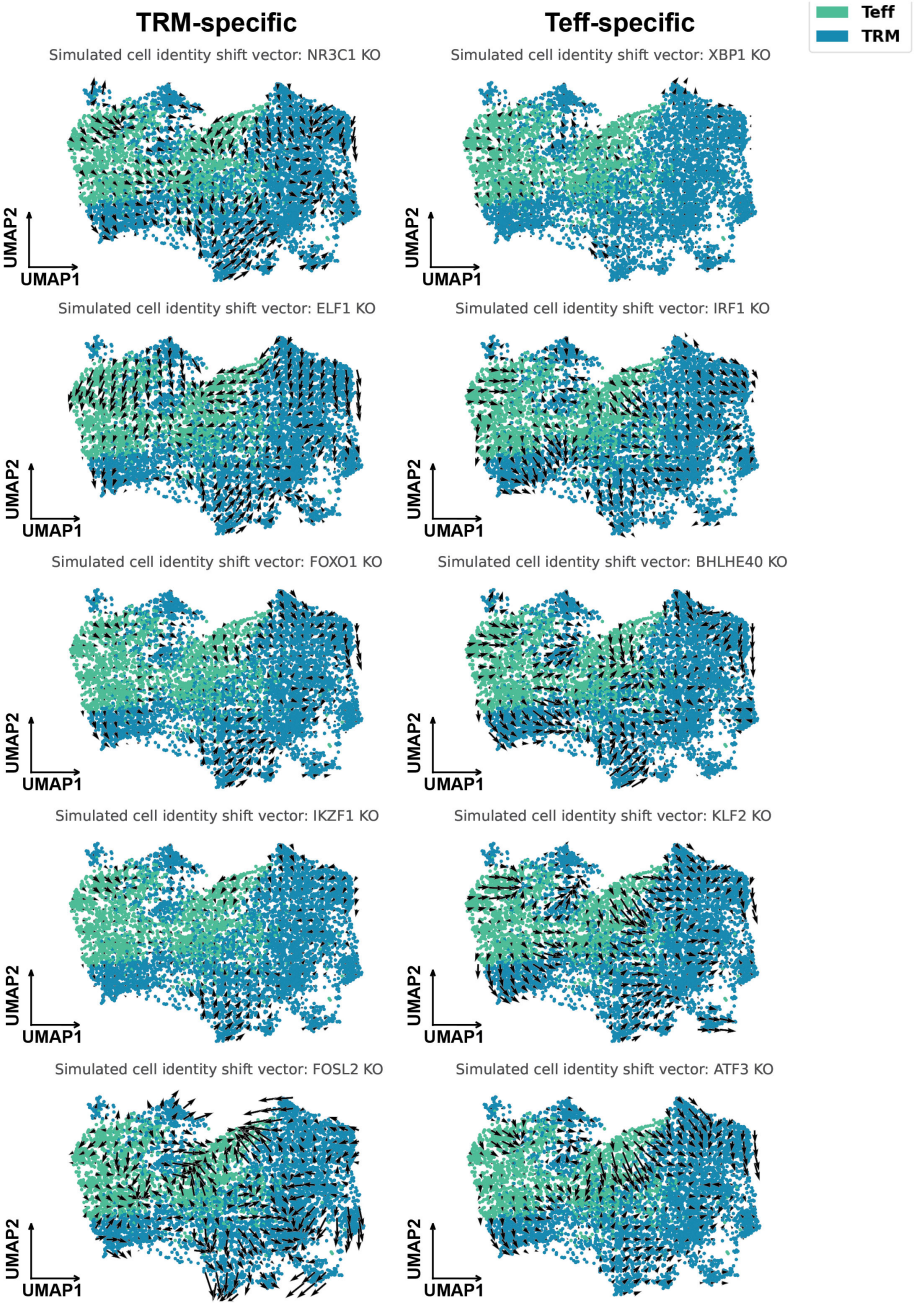
CellOracle predicts the knockout effect of SCENIC-predicted TRM/Teff-specific TFs. UMAPs are colored by tissue-specific cell type. Arrows show Gaussian-smoothed, grid-aggregated transition vectors obtained by projecting each cell’s predicted post-knockout transitions onto its 200 nearest neighbors in UMAP space.

To test whether the SCENIC-inferred cell subtype-specific TFs are functionally linked to cell state, we used CellOracle (38) to simulate *in silico* knockouts (KOs) of the top 5 TFs identified in TRM and Teff cells, since we are mainly interested in promoting the γδ T cells’ effector function. Briefly, CellOracle utilizes reference GRN and then fits regularized linear models to learn context-specific edge weights that predict target-gene expression from candidate TFs. KO effects are simulated by setting the TF of interest to 0 expression, propagating signals through this GRN, and estimating cell-state transition probabilities. Indeed, although with varied effects, it is predicted that by knocking out the top TRM-specific TFs FOXO1, IKZF1, ELF1, and NR3C1, the CRC γδ T cells shift towards the effector state, which is visualized by the general trend of field vectors flow from the cluster of TRM to the cluster of effectors (**Figure 4**). Similarly, by knocking out the top Teff-specific TFs IRF1, BHLHE40, KLF2, and ATF3, the CRC γδ T cells shift towards the TRM state.

All novel TF-target gene pairs were incorporated into the NicheNet gene regulatory network. In the original NicheNet study, model performance was evaluated by predicting ligands from RNA-seq data derived from experimentally perturbed conditions using known ligands, referred to as the “gold standard.” Because γδ T cell-specific additions primarily expand underrepresented signaling and regulatory interactions rather than altering shared core pathways, we expected the model performance on unrelated test data to remain stable. Indeed, benchmarking confirmed that integrating both the SCENIC-predicted GRN and literature-curated γδ T cell ligand-receptor interactions did not compromise the model’s ability to correctly identify perturbed ligands in the reference dataset (**Supplementary Figure S1**).

### NicheNet combined with SCENIC infers ligands including 4-1BBL and IL-15 that shape γδ T cell activation in CRC

**Figure 5:**
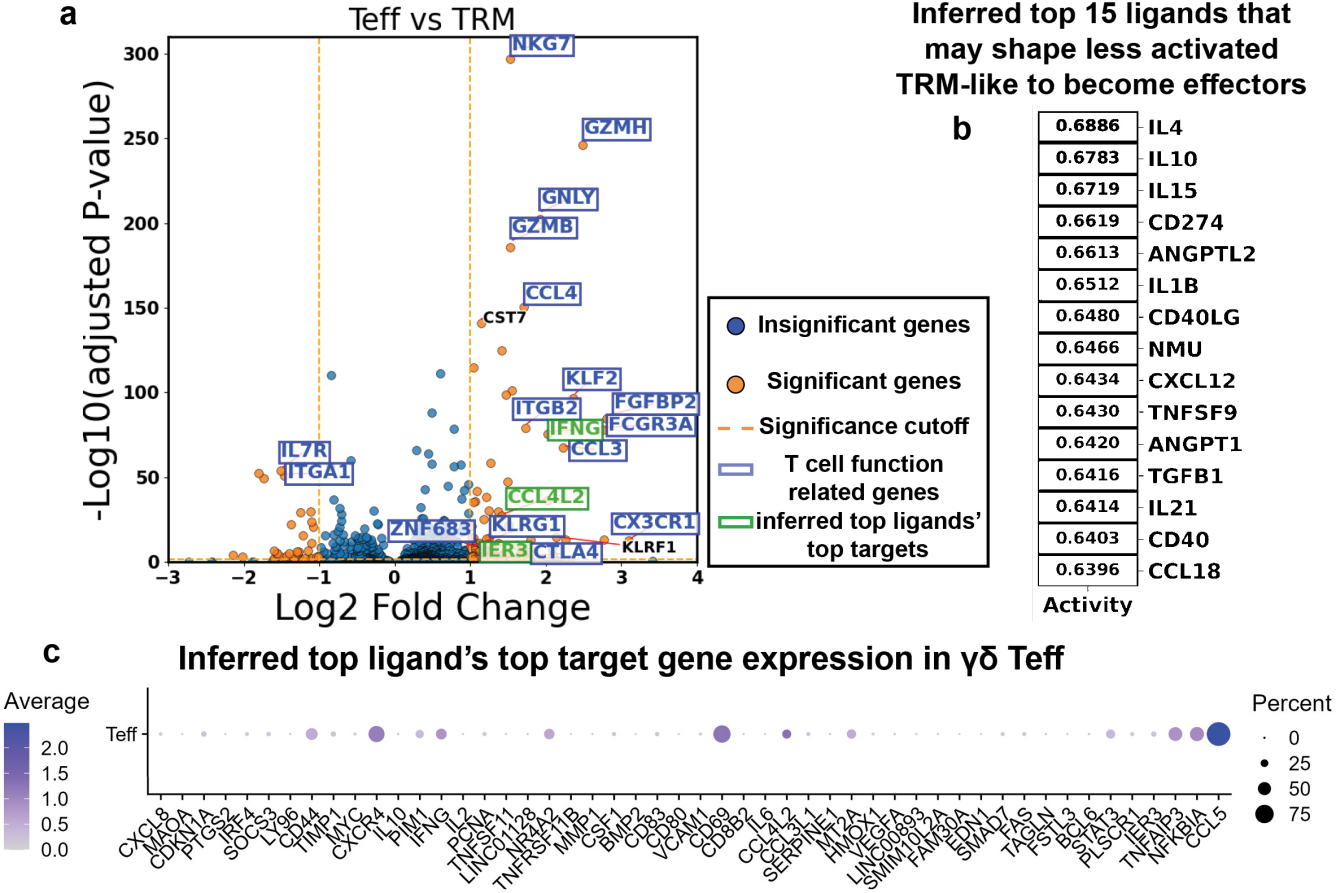
NicheNet predicts ligands that most influence the TRM to Teff transition from differential gene expression. **(a)** Volcano plots showing the differentially expressed genes between Teff and TRM (Teff upregulation on the right side). Each dot is a gene. The X-axis shows the log2 fold change in gene expression between two groups, and the Y-axis shows the -log10 of the multi-comparison-corrected P-value of each gene’s differential expression being statistically significant. **(b)** Top [ranked by area under the receiver operating characteristic curve (AUROC)] 15 NicheNet-inferred ligands that shape the differential upregulation in Teff with respect to the TRM. **(c)** Dot plot showing the expression and fraction of expressing cells of the top 15 NicheNet-inferred ligands’ top 5 target genes collectively in Teff cells.

After customizing the NicheNet model, we computed DEGs between Teff and TRM γδ T cells in our atlas. In addition to classical cytotoxic molecule-encoding genes such as *GZMB*, *NKG7*, and *GNLY*, and effector function-related genes like *IFNG*, Teff cells also upregulated *KLRG1*, *CX3CR1*, and *ZNF683* (encoding Hobit) (**Figure 5a**; full list in **Supplementary Table S2**). Collectively, these DEGs reflected the effector-associated transcriptional profile of Teff cells.

**Figure 6:**
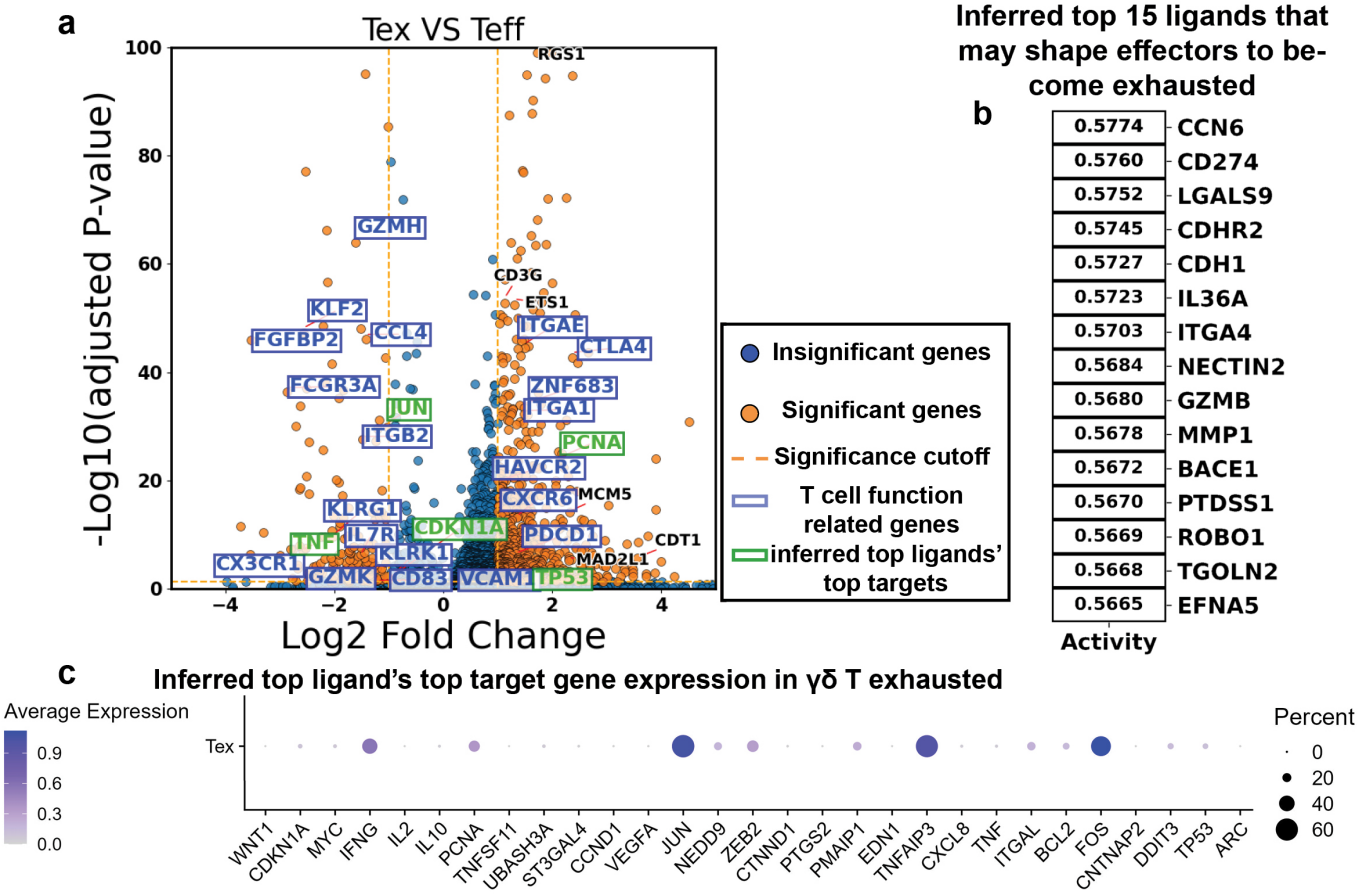
NicheNet predicts ligands that most influence the Teff to Tex transition from differential gene expression. **(a)** Volcano plots showing the differentially expressed genes between Tex and Teff (Tex upregulation on the right side). Each dot is a gene. The X-axis shows the log2 fold change in gene expression between two groups, and the y-axis shows the -log10 of the multi-comparison-corrected P-value of each gene’s differential expression being statistically significant. **(b)** Top 15 NicheNet-inferred ligands that shape the differential upregulation in Tex with respect to the Teff. **(c)** Dot plot showing the expression and fraction of expressing cells of the top 15 NicheNet-inferred ligands’ top 5 target genes collectively in Tex cells.

To identify potential upstream regulators of this transcriptional program, we input the Teff-upregulated genes into NicheNet to infer ligands most likely to drive the TRM-to-Teff transition. Among the top 15 predicted ligands were IL-4, IL-15, IL-21, and TNFSF9 (encoding 4-1BBL), suggesting they may contribute to promoting γδ T cell activation. However, the activation landscape also included immunosuppressive signals, with TGFB1, IL10, and PD-L1 (CD274) predicted as counter-regulatory ligands (**Figure 5b**; list in **Supplementary Table S3**). For comparison, we also used the DEG-based NicheNet approach to predict ligands that potentially drive the transition from Teff to Tex. This analysis identified ligands corresponding to several inhibitory receptors, including CD274 (PD-L1, binding PD-1), LGALS9 (binding TIM-3), NECTIN2 (binding TIGIT), and CDH1 (E-cadherin, binding KLRG1), highlighting their potential roles in promoting effector exhaustion (**Figures 6a, b**; list of DEGs in **Supplementary Table S4**; list of ligands in **Supplementary Table S5**).

Although NicheNet evaluates the full list of differentially expressed genes (DEGs) to generate ligand predictions, we asked which target genes were most strongly associated with the top-ranked ligands. Specifically, we examined the top five target genes for each of the top predicted ligands and assessed their expression in the goal cell states, Teff and Tex, to gain insight into the basis of the NicheNet predictions (ligand-target gene matrices in **Supplementary Figures S2a, b**). Several of the top target genes were indeed highly expressed in goal state cells (**Figures 5c**, **6c**). Among them, *IFNG*, *CCL4L2*, and *IER3* stood out as some of the most upregulated genes in the Teff population (**Figure 5a**).

**Figure 7:**
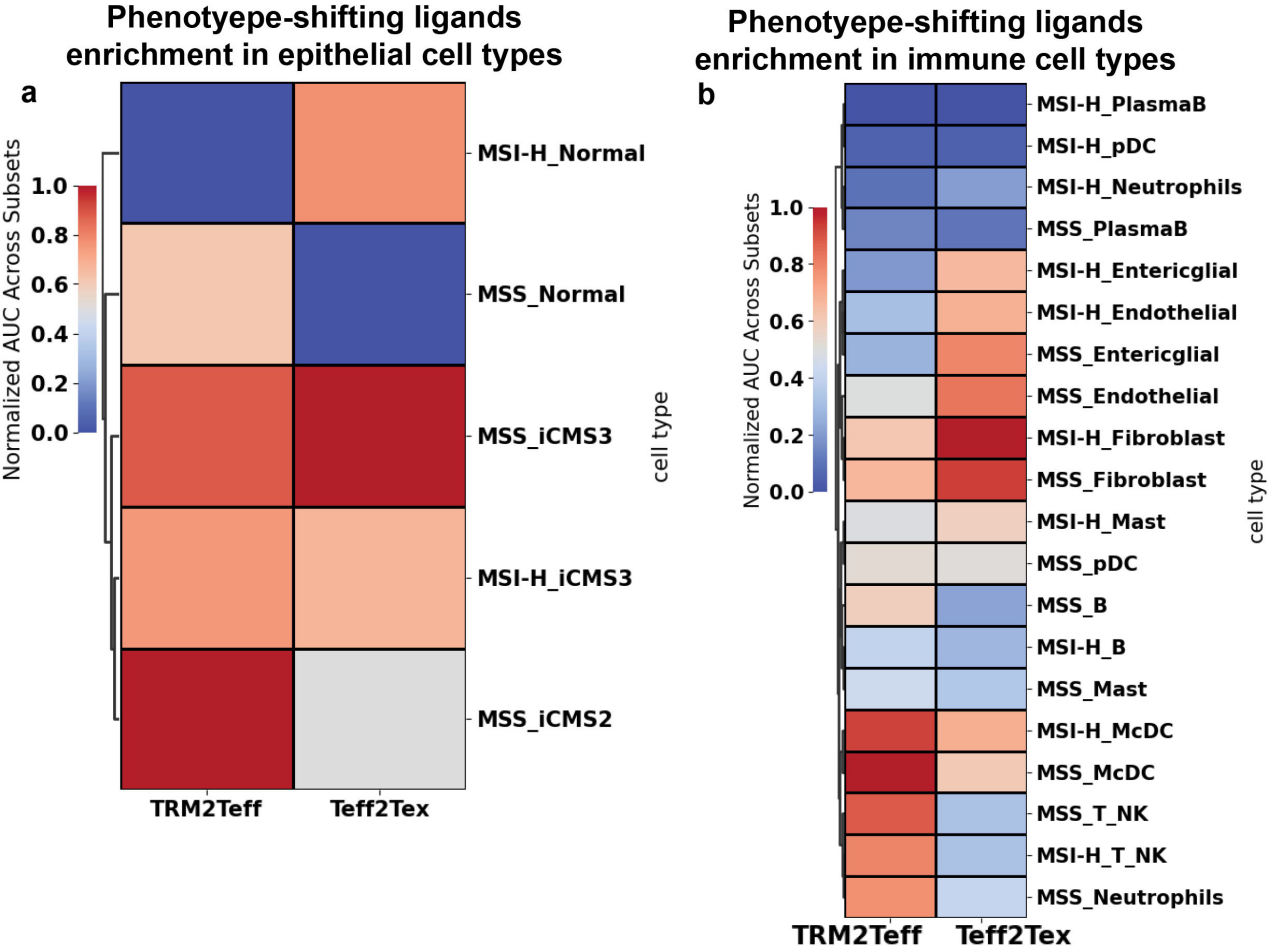
Human CRC γδ T cells phenotype-shifting ligands are enriched in macrophage, dendritic cells, fibroblasts, and iCMS2/3 CRC cells. The enrichment of the NicheNet-inferred phenotype-shifting ligands in each CRC TME **(a)** epithelial **(b)** non-epithelial cell type was quantified by the area under the recovery curve (AUC) against the top 30 (ranked by area under the receiver operating characteristic curve (AUROC), AUROC > 0.5) NicheNet-inferred ligands. Results are visualized by a heatmap, where the color represents the enrichment min-max normalized across cell types. TRM2Teff, TRM to Teff. Teff2Tex, Teff to Tex. MSS, microsatellite stable; MSI-H, microsatellite instability-high.

We also compared the ligand prediction results of the unmodified NicheNet model with those of the γδ T cell-tailored NicheNet. The inclusion of γδ T cell-specific edges did not significantly increase the number of novel ligands predicted to perform better than random (defined as AUROC > 0.5), but it did adjust the relative ranking and confidence of ligand predictions within the γδ T cell context (**Supplementary Figures S3a–c**; full list of ligands predicted by unmodified NicheNet in **Supplementary Tables S6**, **S7**). This refinement can support future experimental designs by prioritizing ligands that are more likely to have a functional impact on γδ T cells. Using the annotated CRC whole-tissue scRNA-seq reference dataset from Joanito et al. (39), we further assessed the enrichment of the top 30 γδ T cell phenotype-shifting ligands across various cell types in the CRC tumor microenvironment. iCMS2-type CRC cells, found in microsatellite-stable (MSS) patients, were enriched for ligands predicted to regulate the TRM-to-Teff transition, whereas iCMS3 cells, also from MSS patients, were enriched for ligands associated with a shift toward exhaustion (**Figure 7a**; detailed expression heatmaps in **Supplementary Figures S4a, b**). In both MSS and microsatellite instability-high (MSI-H) CRC, monocytes/classical dendritic cells (McDCs) exhibited the strongest potential to drive γδ T cell transition from a less activated, TRM-like state to a more activated, effector phenotype (**Figure 7b**; expression heatmap in **Supplementary Figure S5a**). Additionally, enrichment analysis indicated that CRC fibroblasts may contribute to the exhaustion of tumor-infiltrating γδ T cells (**Figure 7b**; detailed ligand expression in **Supplementary Figure S5b**).

### Geneformer predicts that overexpression of *NCR2* and *NKG2E* shifts cell state toward an effector phenotype

Next, we performed *in silico* perturbation on the TRM-like γδ T cell profile using Geneformer to identify which gene overexpression events would most effectively shift the cell state toward Teff. Briefly, Geneformer leverages a BERT-based masked language modeling (MLM) framework trained on over 95 million human single-cell expression profiles to learn gene-gene relationships in diverse biological contexts. This enables the computation of the contextual embeddings of cell states based on their expression profiles. The *in silico* overexpression analysis involves simulating the increased expression of candidate genes within the original TRM cell state and assessing which gene perturbations produce a cell embedding that is similar to the embedding of the goal state, in this case, Teff (**Figure 2**).

**Figure 8:**
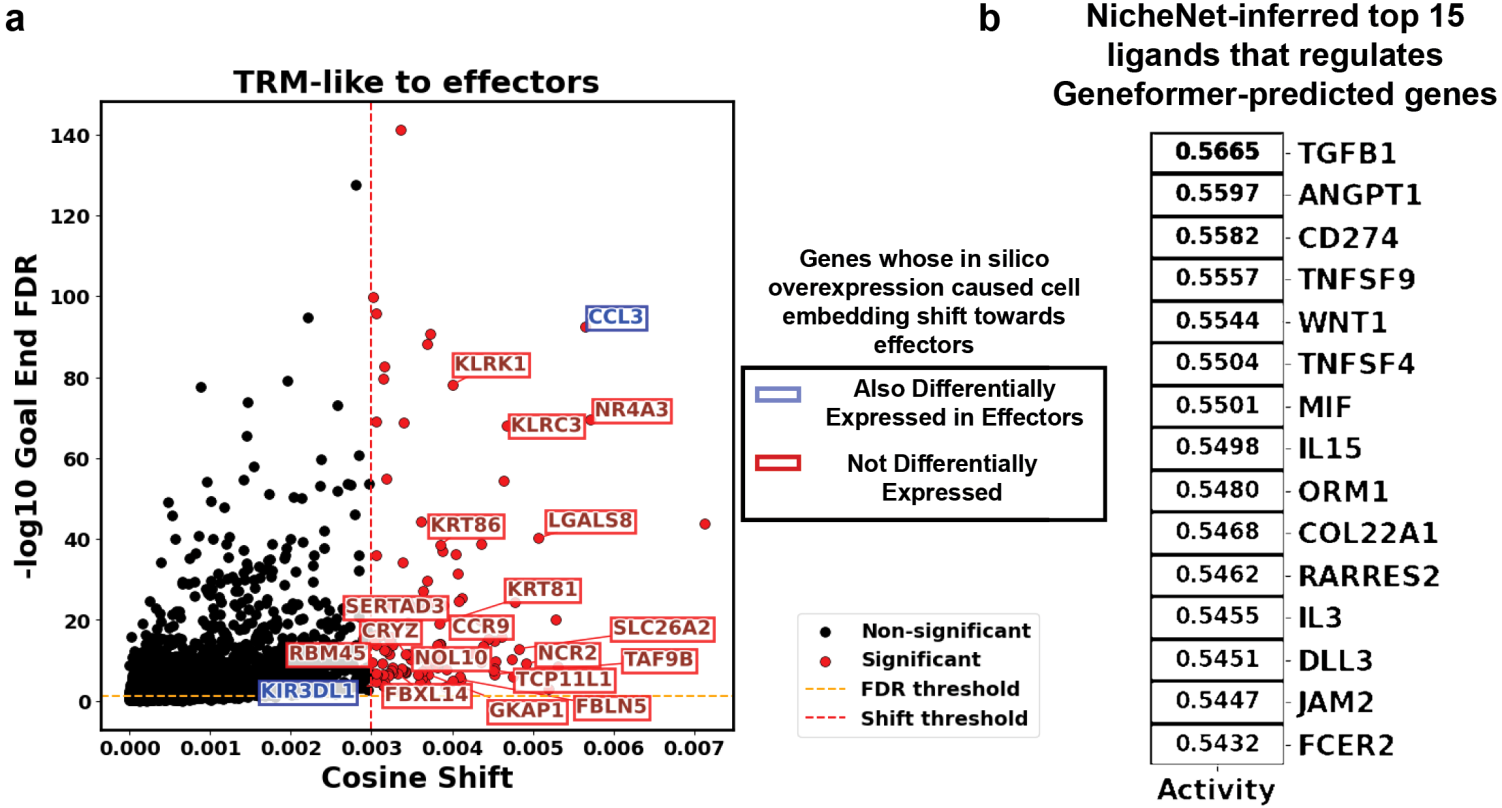
*In Silico* perturbation using geneformer. **(a)** Scatter plot visualizing the cosine shift caused by the *in silico* overexpression of each gene from the TRM state towards the Teff state. The X-axis shows the cosine shift, and the Y-axis shows the multi-comparison-corrected P-value of each gene being statistically significant. **(b)** Top 15 NicheNet-inferred ligands (derived from Geneformer-predicted state) that shape the differential upregulation in Teff with respect to the TRM.

Using the fine-tuned Geneformer model, which achieved 88% accuracy in classifying TRM and Teff labels (**Supplementary Figure S6**), we identified several genes whose in silico overexpression was associated with a shift in embedding toward an effector-like state. These included *CCR9*, *NCR2*, *KLRK1* (NKG2D), *KLRC3* (NKG2E), *NR4A3*, and *MMP9* (**Figure 8a**; full list of DEGs in **Supplementary Table S8**). Notably, most of these genes were not differentially expressed between Teff and TRM-like cells, indicating that Geneformer uncovered novel perturbation targets rather than simply retrieving genes whose differential expression reflects the Teff-like transcriptional profile. We then re-applied the γδ T cell-refined NicheNet to the set of Geneformer-predicted perturbation genes to infer upstream ligands. Among the top predicted ligands were IL-15 and TNFSF9, which also ranked highly in the initial NicheNet analysis based on differentially expressed gene signatures (**Figure 8b**; full list of ligands in **Supplementary Table S9**).

## Discussion

In this study, we developed a γδ T cell-tailored ligand inference framework to investigate the molecular drivers of γδ T cell activation in CRC. In our previous study, by integrating multi-study single-cell RNA-seq data, we constructed a comprehensive atlas of CRC-infiltrating γδ T cells and identified major subsets, including TRM-like bystanders and cytotoxic effectors. To uncover signals responsible for driving transitions between these phenotypes, we combined differential gene expression analysis, SCENIC-inferred gene regulatory networks, NicheNet ligand inference, and the large language model Geneformer for in silico perturbation. We refined the NicheNet framework by incorporating γδ T cell-specific ligand-receptor interactions and transcriptional regulatory relations. This approach revealed key ligands, including IL-15 and TNFSF9, as potential regulators of γδ T cell activation. Notably, Geneformer identified perturbation targets such as NCR2 and KLRC3 (NKG2E), which were not differentially expressed but capable of inducing a Teff-like state, highlighting the importance of natural killer receptor signaling other than the well-known NKG2D and NCR3. Together, our framework provides a systems-level perspective on the extrinsic and intrinsic factors shaping γδ T cell phenotypes in CRC and offers a prioritized list of experimentally testable candidates for immunomodulation.

SCENIC revealed distinct TFs that regulate γδ T cell subtypes in human CRC. MYBL2, associated with cell proliferation (40), may indicate a proliferative potential in γδ T cells expressing exhaustion markers. SPI1 acts downstream of PRDM1 to enhance PD-L1 transcription (41), indicating a regulatory role in the Tex γδ T cell subset. The CellOracle in silico perturbation further supports SCENIC’s prediction that different CRC-infiltrating γδ T cell states are regulated by distinct TFs. TRM is predicted to be most specifically regulated by NR3C1, ELF1, FOXO1, IKZF1, and FOSL2. Teff is predicted to be regulated by XBP1, IRF1, BHLHE40, KLF2, and ATF3. Indeed, literature-reported functions of these subset-specific TFs align well with the observed activation states. TFs enriched in the less-activated TRM subset were associated with promoting cell survival, preventing exhaustion, and suppressing effector function, whereas TFs enriched in the effector subset were primarily involved in active interferon production and signaling and in stress responses. NR3C1 encodes the glucocorticoid receptor (GR), a ligand-activated TF that mediates cortisol’s immunosuppressive effects. FOXO1 maintains long-lived cells; AKT phosphorylates FOXO1, excluding it from the nucleus and thereby relieving its restraint on effector differentiation, which is consistent with the higher IL7R levels we observe in TRM (42). A study combining mouse transcriptomics and epigenomics reported that FOXO1 is required to sustain TRM in the small-intestinal lamina propria and in the colon (43). FOXO1 also antagonizes IL-17 programs, which may explain our earlier observation that human CRC γδ T cells do not produce IL-17 (44). IKZF1(Ikaros), previously shown to repress gene programs associated with type I interferon responses (45), may help explain the limited effector function observed in TRM-like γδ T cells. For FOSL2, chronic-stimulation experiments in mice indicate that miR-155 promotes T cell exhaustion in part by downregulating FOSL2, which then reduces AP-1 activity and permits an “exhausted” gene program (46). KLF2, whose downregulation is required for the establishment of tissue residency (47), promotes S1PR1 expression, facilitating T cell circulation through the bloodstream and lymphatic system (48–50). It has also been implicated in enhancing effector function and preventing exhaustion in CD8^+^ T cells during LCMV infection (51), raising the possibility that KLF2 may influence effector function or exhaustion dynamics in γδ T cells. IRF1 is required for optimal granzyme B and perforin expression and for responsiveness to IL-12/IL-18 signaling in CD8 T cells (52). BHLHE40 responds to activation and inflammation, and human T cells edited to lack BHLHE40 show increased IL-10 and reduced IFN-γ, whereas BHLHE40 overexpression boosts IFN-γ secretion (53).

Our DEG analysis highlights key features of γδ T effector cells in human CRC. *KLRG1*, a marker of terminal differentiation and senescence in CD8^+^ effector T cells (54, 55), was highly expressed in γδ Teff cells in our integrated dataset. As an inhibitory receptor, KLRG1 impairs T cell effector function by blocking AKT phosphorylation through the recruitment of phosphatases that convert PIP3 to PIP2 (56). In murine models, KLRG1 inhibition enhances antitumor activity (57). Flow cytometry studies in human colorectal cancer have shown that KLRG1*^+^* T cells exhibit impaired cytotoxicity (58); however, the functional consequences of KLRG1 expression in γδ T cells remain unclear. It is yet to be determined whether KLRG1 dampens γδ T cell function as it does in natural killer (NK) cells (59), or whether its expression is more permissive, as has been debated for PD-1, which may not signify exhaustion in γδ T cells but instead reflects an activation state (60).

Interestingly, KLRG1^+^ CD8^+^ Teff cells have been shown to downregulate KLRG1 and differentiate into memory subsets (61). KLRG1^+^ γδ T cells are also found in CMV-uninfected newborns (62) and become more prominent following CMV infection and in adulthood (63). In murine cytomegalovirus (MCMV) infection, KLRG1^+^CX3CR1^+^ γδ effector memory cells populate lung and liver vasculature and mount strong antiviral responses (64). Although a scRNA-seq analysis of CRC patient samples from TCGA reported enrichment of KLRG1^+^ cytotoxic T cell signatures associated with favorable outcomes (65), it may be premature to attribute a positive role to KLRG1 in antitumor immunity.

Additionally, ZNF683 (Hobit), which has been implicated in promoting IFNG transcription in effector T cells (66) and marks precursors of tissue-resident memory T cells (67), was also upregulated in γδ Teff cells, supporting their cytotoxic potential and tissue-localized phenotype. Compared to the less activated TRM-like population, Teff cells also showed downregulation of *IL7R* and *ITGA1*, suggesting reduced emphasis on survival and increased migratory capacity.

Ligand prediction by NicheNet in the TRM-Teff transition provided many interesting targets for γδ TCR-independent activation. IL15, needless to say, is a well-studied ligand essential for T cell survival, proliferation, and effector function (68). It has been used in multiple studies to expand Vδ2^+^ γδ T cells or CAR γδ T cells and enhance their cytotoxicity (69–71), although there are also reports that continuous IL-15 signaling leads to exhaustion in NK cells (72, 73). Another interleukin in the list, IL-21, is also known to promote T cell proliferation and cytotoxic differentiation (74). Literature has reported its effect on providing short-term proliferation (75) and enhancing the antitumor cytotoxic response in γδ T cells in the presence of IL-2 (76). Studies have also shown IL-21’s synergistic effect with IL-15 in promoting CAR T and NK cell expansion (77, 78). Interestingly, both CD40 and its ligand, CD40LG, were among the top ligand list. Recent findings show that CD40LG expressed on CD8^+^ T cells can engage CD40 on dendritic cells, leading to their activation and promoting CD8^+^ T cell effector differentiation (79). The CD40LG on CD8 T cells is found to interact with CD40 on dendritic cells, such that this interaction not only promotes CD8 T cells’ effector function (80) but also the survival of dendritic cells in antitumor response (81). In mice parasite infection, γδ T cells enhance dendritic cells activation through CD40LG-CD40 interactions (82). Considering that TRM-to-Teff NicheNet-predicted ligands were most enriched in McDCs in Joanito et al.’s dataset, a model where γδ T cells promote dendritic cell activation via CD40LG*-*CD40 interactions, thereby reinforcing their own effector programming, is a reasonable hypothesis. Indeed, although γδ cells are known for MHC-independent activation, our result of McDCs being identified as the top candidate influencing γδ cells’ effector differentiation encourages future studies that focus on antigen-presenting cells (APC)-γδ cells interactions.

On the other hand, there is also a possibility that CD40 is expressed on γδ T cells and interacts with CD40LG^+^ cells in the TME. The expression of CD40 on activated CD8^+^ T cells has been reported (83, 84), and *in vitro* stimulation of CD40^+^ human CD8^+^ T cells with CD40LG^+^ artificial APCs increased both the total number and percentage of effector cells (84). Although such CD40 expression appears to be transient, it may still exert long-term effects on T cell differentiation, at least in terms of cell numbers, as pointed out in a previous study (84).

Another interesting finding is the presence of TNFSF9, the 4-1BB ligand, among the top Teff-shifting ligands, rather than CD80/86, which bind CD28. In T cells, TNFSF9 provides a potent co-stimulatory signal through 4-1BB, amplifying the proliferation and survival of effector T cells (85). Since the second generation of CAR-T therapy, the intracellular domain of 4-1BB has been incorporated into the CAR design (86) to achieve a better activation. Current CAR γδ T cells typically use Vδ2 TCRs, the most abundant γδ T subset in blood, and incorporate the CD28 (87) signaling domain based on second-generation CAR αβ T cell design (14). However, whether CD28 effectively helps the activation of most intraepithelial γδ T cells, or generally speaking, the Vδ1 subset, which has stronger cytotoxicity (88), tissue residency (89), and tumor infiltration (90–92), remains unclear. Our preliminary scRNA expression data of intraepithelial γδ T cells in healthy human colon (93) agrees with the previous mouse (94) and human (95) observations that they do not express CD28. Additionally, there are contradictory results of the CD28 antibody’s effect on human γδ T cells (96, 97). There are multiple potential therapeutic co-stimulatory receptors, like 4-1BB (98), CD27 (99), and CD6 (100), but stimulating them in the CRC settings has unknown results, plus there could be more undiscovered. The identification of 4-1BBL among the top ligands but not CD80/86 suggests that 4-1BB is likely to serve as a more prominent or contextually relevant co-stimulatory signal for γδ T cells in human CRC. Indeed, ligand prediction based on Geneformer-derived overexpression targets also supported the importance of 4-1BBL.

The Geneformer-identified perturbation targets are also very interesting. The NKG2D and NCR3 have been relatively well discussed in γδ T cells, but our analysis also highlights the NCR2 and NKG2E. Unlike conventional αβ T cells, γδ T cells can upregulate NCR2 (NKp44) upon activation. Resting peripheral γδ T cells lack NKp44, but cytokine-driven stimulation induces its expression in a subset of cells (101). Notably, NKp44 induction is largely restricted to Vδ1^+^ γδ T cells. Prolonged TCR stimulation (more than 2 weeks) in the presence of IL-15 or IL-2 leads to robust NKp44 (and NKp30) expression in Vδ1^+^ cells, whereas Vδ2^+^ cells show minimal NKp44 upregulation (102, 103). KLRC3, alias NKG2E, is an activating NK receptor that is closely related to the well-known NKG2C. Both NKG2C and NKG2E form heterodimers with CD94 and signal through the DAP12 adapter, analogous to NK cells (104). CD94-NKG2C recognizes HLA-E and signals through the ITAM adaptor DAP12, recruiting Syk/ZAP-70 to promote cytotoxicity (105).

Admittedly, our pipeline relies on the sequential application of several tools (SCENIC, NicheNet, and Geneformer), which may introduce compounded modeling uncertainty. We use these tools not as definitive sources of truth but as complementary, mechanistically interpretable aids for prioritizing hypotheses, especially in the context of rare cell types such as γδ T cells, where validated PPI and TF-target data are scarce. In this way, inputting predictions from one tool into another represents a practical way to take cell type specificity into consideration in a time- and cost-efficient manner. Nonetheless, we expect that as γδ T cell-specific interaction data are experimentally validated, our framework can be progressively updated and benchmarked against these ground truths. Likewise, if ChIP-seq data for all TFs predicted by SCENIC become available in CRC γδ T cells, the verification shown in **Figure 3**, which currently relies on ChIP-seq data from general T cells and NK cells in public databases due to resource limitations, would be more convincing. An additional limitation is that we did not yet explicitly account for the heterogeneity in sex, age, and anatomical location present in the integrated single-cell datasets, and future extensions incorporating bootstrapping and subgroup modeling will be required to systematically evaluate its impact on γδ T cell activation states and ligand associations.

In this current study, we focused on genes upregulated in Teff versus TRM as the NicheNet gene set of interest and on overexpression-like perturbations from Geneformer. This activation-centric design matches the default pySCENIC output of positive regulons and mitigates some of the ambiguity introduced by dropout when interpreting downregulation in scRNA-seq data. The use of positive DEGs as the input for NicheNet is also practiced by single-cell best practices (106). Nonetheless, future extensions of our framework could explicitly incorporate downregulated genes and knockdown-like perturbations to identify ligands that stabilize TRM-like programs or repress effector differentiation.

For ligand inference, several other tools are also available. For example, scMLnet (107) reconstructs a multilayer signaling network by integrating prior knowledge with single-cell expression data. It first defines a ligand-receptor subnetwork by selecting highly expressed ligands in the sender population and cognate receptors in the receiver population, then builds a TF-target subnetwork in the receiver by testing enrichment of known TF-target relationships among highly expressed genes to infer activated TFs. These activated TFs are then connected back to upstream receptors using curated receptor-TF interactions, and the ligand to receptor, receptor to TF and TF to target subnetworks are merged into a cascade that is further refined using receptor-TF and TF-target expression correlations. Similarly, scSeqComm (108) combines statistical and network-based steps: it computes an intercellular signaling score for each candidate ligand-receptor pair from cluster-level expression, and then an intracellular signaling score that quantifies how strongly downstream signaling pathways and TF regulons linked to the receptor are activated in the receiver cells, based on curated signaling-pathway and transcriptional-regulatory networks. Conceptually, both scMLnet and scSeqComm share NicheNet’s reliance on curated ligand-receptor and regulatory knowledge, but they differ in how they score ligand-target connections: NicheNet propagates ligand signals over an integrated prior network using a Personalized PageRank-based scheme, whereas scMLnet and scSeqComm explicitly prioritize the ligand-target chain using dataset-specific statistical evidence.

To validate the predicted ligand-receptor interactions, we propose complementary spatial and perturbation approaches. Ideally, spatial transcriptomics and multiplexed protein imaging would be used to confirm that γδ T cells and ligand-expressing cells are in direct spatial proximity. As a computational alternative, γδ T cell transcriptional profiles can be mapped onto CRC spatial transcriptomics sections (for example, Visium) using tools such as Tangram (109). One can then quantify whether predicted ligand-expressing cells or niches are enriched for γδ T cell probability using spatial cross-correlation metrics and local Moran’s I, and compare the observed co-localization to null distributions generated by spatially constrained spot permutations or block randomization within tissue regions. In parallel, pooled CRISPR Perturb-seq in primary tumor-infiltrating γδ T cells can be used to test causality. An sgRNA library would target receptors prioritized by this study and key downstream signaling nodes, including non-targeting and pathway-positive controls. Transduced cells would be cultured in CRC tissue-conditioned medium whose cytokine and ligand composition is quantified by a multiplex platform, then profiled by single-cell RNA-seq with guide calling and, optionally, CITE-seq antibodies for Teff, TRM, and exhaustion surface markers.

Several emerging immunotherapies, including immune checkpoint blockade, CAR T cell therapy, and bispecific T-cell engagers, are being actively explored to enhance anti-tumor immune responses. Immune checkpoint inhibitors (e.g., antibodies targeting PD-1) have achieved durable responses in the subset of CRC patients with mismatch-repair deficient, microsatellite instability-high (MSI-H) tumors, leading to regulatory approvals for this population (110). Chimeric antigen receptor (CAR) T cell therapy is also being pursued in CRC by engineering patient T cells to recognize tumor-associated antigens, and early-phase trials have reported initial safety and antitumor activity with this approach, even though CAR T strategies for CRC remain in early development (111). Another innovative strategy involves bispecific antibodies. These agents are often formatted as bispecific T-cell engagers, and they bind simultaneously to a tumor antigen and to the CD3 complex on T cells, physically linking immune cells to cancer cells and triggering tumor cell lysis. Prototypes targeting CRC antigens like EGFR or carcinoembryonic antigen have demonstrated potent tumor cell killing in preclinical models and even in patient-derived samples (112). Recent advances in γδ T cell-based immunotherapy, including CAR γδ T cell applications in hematological malignancies and solid tumors, have been comprehensively reviewed by Dong et al. (14), providing important clinical and translational context for our findings.

Together, these findings highlight ligands such as IL-15 and TNFSF9 as promising candidates for promoting γδ T cell activation in CRC. In addition, NCR2 and NKG2E emerged as potential receptors through which γδ T cells may recognize CRC. These insights can inform the design of engineered γδ T cell therapies aimed at enhancing antitumor function and supporting both early tumor elimination and long-term postsurgical surveillance.

## Method

### Data acquisition

The human CRC-infiltrating γδ T cell scRNA-seq dataset was curated as described in our previous study (22). Briefly, we searched for publicly available scRNA-seq datasets from treatment naïve CRCs in indexed journals and Gene Expression Omnibus. In each study, cell clusters that highly co-expressed CD3D, CD3E, CD3G, CD247, and TRDC were identified as γδ T cells. Distinct γδ T cell clusters with more than 50 cells were identified from 9 studies that provided raw counts. Human CRC whole-tissue scRNA-seq datasets were obtained from Gene Expression Omnibus (GEO) Series GSE178341 (113), GSE200997 (114), GSE245552 (115), GSE231559 (116), GSE188711 (117), GSE161277 (118), GSE108989 (119), GSE178318 (120), and PubMed PMID35773407 (39). The datasets analyzed in this study were derived from a diverse cohort of patients, encompassing different sexes, anatomical sites, demographic backgrounds, and disease stages, as detailed in our previous work. Since this study involved secondary analysis of publicly available scRNA-seq datasets, no additional group allocation or randomization was performed by the authors. Investigators were not blinded during data analysis, as all datasets were de-identified and publicly accessible. No power calculations were conducted, as sample sizes were determined by the availability and characteristics of the original datasets. Detailed experimental protocols and patient recruitment information can be found in the original publications associated with each dataset.

### Data processing

For processing the original dataset, only cells in the colon mucosa were kept. If processed data is not provided, Seurat (121) and Scanpy (122) were used for downstream analysis. For quality control, low-quality cells were dropped based on their low UMI counts (<500), high mitochondrial gene counts (>20%), and a low number of uniquely expressed genes (<200). If the unprocessed data of a study has multiple samples, data integration was done by using Seurat on the normalized and log-transformed raw counts, which means each cell’s counts are divided by its total counts, multiplied by 10,000, then log‐transformed. SCTransform was applied to raw counts of γδ T cells isolated from each study. As we have briefly summarized in the previous study, raw counts were fitted into negative binomial distributions whose expectation was a function of the total count of cells. The coefficients of the gene’s general linear model are further regularized by the kernel regression with the coefficients of other genes that have similar average expression. Regressed gene expression calculated from the regularized generalized linear model coefficients was referred as “SCTransform-corrected counts,” and log-transformed SCTransform-corrected counts were used for later visualization. The Pearson residual of a cell’s observed gene expression to the SCTransform-corrected counts was used for integration and later performing principal component analysis (PCA, 50 pcs kept). Leiden clustering was performed based on the computed neighborhood graph of observations (UMAP, 50 pcs, size of neighborhood equals 15 cells) to reveal the general subtypes. In order to delineate cell subtypes, further subclustering was performed on each subcluster at a resolution range from 0 to 1 (detailed resolution for each step of subclustering was recorded in the published code). The cell type annotating strategy was detailed in our previous study. In short, we identified four distinct subtypes of γδ T cells in our integrated dataset: poised effector-like T cells (Teff, markers: *KLF2, KLRG1*, *TBX21*, *IFNG* and low *IL7R, ITGAE, ITGA1, CCR7, SELL*), tissue-resident memory T cells (TRM, markers: *ITGAE, ITGA1* and low *CCR7, SELL, KLF2*), progenitor exhausted T cells (Tpex, markers: *TCF7, PDCD1, CTLA4, LAG3, TIGIT, HAVCR2*), and Tex (*GZMB*, *PDCD1, CTLA4, LAG3, TIGIT, HAVCR2* and no *TCF7*). Effector and exhaustion scores were calculated for each cell based on the aforementioned genes using the scanpy function score_genes against the same number (6) of random genes.

For data analysis, the tools we used are detailed below. We also summarize the inputs and outputs of these tools in **Supplementary Table S9** to help readers conveniently follow our workflow*.SCENIC*: Cell expression profile of the tumor-infiltrating γδ T cells was input into the SCENIC (v1.3.1) pipeline using default parameters. List of TFs “hs_hgnc_curated_tfs.txt,” motifs “motifs-v10nr_clust-nr.hgnc-m0.001-o0.0.tbl,” and the motif ranking databases “hg38_500bp_up_100bp_down_full_tx_v10_clust.genes_vs_motifs.rankings.feather” used for the pipeline were obtained from https://resources.aertslab.org/cistarget/, http://github.com/aertslab/pySCENIC/blob/master/resources/hs_hgnc_curated_tfs.txt, and https://resources.aertslab.org/cistarget/motif2tf/. As indicated in the file name, our motif search range is 500 bp upstream and 100 bp downstream of the target gene TSS. The output regulons from the cisTarget are integrated into the NicheNet’s network. Briefly, these regulons are considered statistically significant. SCENIC calculates the enrichment of a TF’s motif in its potential target genes from their gene vs. motifs ranking matrix. First, by default, cisTarget keeps the top 75% of the regulon members (target genes) of a motif by the motif’s enrichment in the gene’s promoter region. Second, a recovery curve for the motif is established by the percentage of inferred target genes recovered from parsing down the ranked list. This area under the curve (AUC) will be normalized over the AUC of all motifs to recover the same set of target genes (i.e., the inferred target genes of the motif) from all genes to obtain the Normalized Enrichment Score (NES), and a motif with NES > 3 by default is considered significant to regulate the inferred target set. For the ChIP-seq verification of the SCENIC prediction, the chromosome coordinates were obtained from Ensembl “Homo_sapiens.GRCh38.113.chr.gtf.gz.” The ChIP-seq data were obtained from ChIP-atlas’ “Homo Sapiens ChIP: TFs and others” category, with “T cells.” “Th1 Cells,” “Th17 Cells,” “CD8+ T cells,” “memory T cells,” “Natural Killer T cells,” “Th2 Cells,” “CD4+ T cells,” and “Natural Killer cells” are being selected as cell types. GenomicRanges was used to simply find the overlap between the +/- 5kb area around the target gene TSS and the peak of the TFs, which are in both SCENIC predictions and the ChIP database. The percentage of a TF’s target genes that could be found at least 1 peak of that TF around its TSS in all obtained ChIP-seq data was visualized. Regulon specificity score of TF in cell subsets is calculated using the pySCENIC function “plot_rss.” Briefly, in SCENIC, the regulon specificity score quantifies how specifically a TF regulon is active in a given cell type. Briefly, SCENIC first ranks gene expression in each cell and uses AUCell to compute an AUC score per regulon per cell, reflecting how well the regulon’s target genes are recovered in that cell. For a given cell type X, two vectors are then defined: P, the distribution of AUC values for that TF’s regulon across all cells, and Q, a binary vector indicating whether each cell belongs to cell type X or not. The RSS is defined as 1 – JS(P||Q), where JS denotes the Jensen-Shannon divergence to describes the divergence of 2 distribution. Higher RSS values indicate that the regulon’s activity is more specifically enriched in that cell type.

### CellOracle in silico perturbation

We used the prebuilt promoter-based human base GRN hg19_gimmemotifsv5_fpr2 and the authors’ recommended automated threshold (123) to construct GRN models, then performed *in silico* KO with default settings (perturbation: set TF expression to 0; n_propagation = 3 to compute post-KO expression; n_neighbors = 200 to estimate transition probabilities). For visualization, all quiver/vector-field plots were drawn with a common scale.

### NicheNet

NicheNet (25) (v2.2.0) is used for ligand inference. The integrated ligand-receptor pairs, signaling network, and gene regulatory networks (prefix 21122021) were obtained from the package’s repository. TF-target gene pairs that are not in NicheNet’s original network and γδ T cell-specific ligand-receptor pairs (CD247 and its ligands EPHA2, EPCR, BTN2A1, BTNL3, BTNL8, CD1B, CD1D, MR1, CD1A, CD1C) were added to the networks with unoptimized weights of 1, given the relatively small number of edges being added and the original author’s comments on the robustness of NicheNet without weight optimization. Functions “construct_weighted_networks,” “apply_hub_corrections,” and “construct_ligand_target_matrix” from the NicheNet package were used sequentially to construct the new ligand-target matrix following NicheNet’s user guide with default parameters. The hyperparameters used during construction were the same as those of the unmodified NicheNet, which was obtained from the package’s repository under the name “hyperparameter_list.rda.” Function “evaluate_model” from the NicheNet package was used to evaluate both the unmodified ligand-target matrix and the new ligand-target matrix’s performance on the “gold standard” dataset that the NicheNet authors used.

The application of the NicheNet is similar to and adapted from our previous study (93). NicheNet was supplied with DEGs and Geneformer. DEGs were calculated by using Scanpy’s tl.rank_genes_groups, Wilcoxon method. DEGs with adjusted p-values < 0.05 and log_2_ fold changes greater than 1 were used as input for NicheNet. Genes expressed in at least 1% of the cells in the receiver subsets (goal states, Teff in the TRM to Teff transition, and Tex in the Teff to Tex transition) were considered as background. Potential ligands were defined as ligands of NicheNet-documented receptors whose encoding genes were expressed in at least 1% of the cells. The top 15 ligands with an AUROC > 0.5 were reported in the main figure, while the top 30 were used for quantifying their enrichment in neighboring cell types. If fewer than 15 ligands met the AUROC > 0.5 threshold, all qualifying ligands were visualized and included in the enrichment analysis.

CRC TME cell type profiles were obtained from Joanito et al. (39). The datasets, epithelial and nonepithelial, had already been quality controlled by the providers. Basic pre-processing, including normalization and log transformation, was done by using Scanpy’s normalize_total and log1p functions. Cells labeled “LymphNode” and “Normal” were excluded. AUC calculations were performed after averaging gene expression by cell type, which is annotated by the original authors. A gene was included in the averaged profile only if its average expression was not less than 0.001 to reduce noise. For each cell type, we iterated down the ranked gene list (ranked by their average expression in the given cell type, descending order) to recover NicheNet-inferred ligands, stopping when encountering a zero-expression gene. The final area under the curve was computed using the auc function from sklearn.metrics.

### Geneformer

Geneformer (26) (v0.1.0) was used to perform in silico overexpression. Data that only contained TRM and Teff was tokenized using Geneformer’s TranscriptomeTokenizer, retaining protein-coding and miRNA genes by default. The pre-trained model “gf-12L-95M-i4096” was fine-tuned by an input data cell type label classification task, using the function “Classifier.validate” with 100 hyperparameter optimization trials, with a train-valid-test split of 0.6-0.2-0.2, max_ncells=None, freeze_layers = 6. The model with the highest accuracy was selected, and its performance as a classifier was visualized using the function “plot_conf_mat.” The initial state (TRM) and the goal state (Teff) were represented by the exact mean [CLS] token embeddings of cells in each group. In silico overexpression was performed using the function “InSilicoPerturber” with max_ncells=5000 and emb_layer=-1. The statistical test of the perturbation result is computed using the function “InSilicoPerturberStats” under the mode “goal_state_shift.” Briefly, in Geneformer, significance was assessed using Wilcoxon rank-sum tests, comparing shifts induced by each gene against the background distribution of all perturbations, with Benjamini-Hochberg correction. Genes with adjusted *p* < 0.05 are considered statistically significant, and a mean cosine shift > 0.003 (empirically selected to reduce background noise) were prioritized. Genes with more than 50 detections in the initial state cells (N_Detections > 50) were reported.

## Data Availability

The datasets presented in this study can be found in online repositories. The names of the repository/repositories and accession number(s) can be found below: https://www.ncbi.nlm.nih.gov/geo/, GSE178341 https://www.ncbi.nlm.nih.gov/geo/, GSE200997 https://www.ncbi.nlm.nih.gov/geo/, GSE245552 https://www.ncbi.nlm.nih.gov/geo/, GSE231559 https://www.ncbi.nlm.nih.gov/geo/, GSE188711 https://www.ncbi.nlm.nih.gov/geo/, GSE161277 https://www.ncbi.nlm.nih.gov/geo/, GSE108989 https://www.ncbi.nlm.nih.gov/geo/, GSE178318 https://pubmed.ncbi.nlm.nih.gov/, PMID35773407.

## References

[1] World Health Organization . Cancer. Geneva, Switzerland: World Health Organization (2025). Available online at: https://www.who.int/news-room/fact-sheets/detail/cancer (Accessed January 7, 2026).

[2] Keum N Giovannucci E . Global burden of colorectal cancer: emerging trends, risk factors and prevention strategies. Nat Rev Gastroenterol Hepatol. (2019) 16:713–32. doi: 10.1038/s41575-019-0189-8, PMID: 31455888

[3] Curry WJ Lengerich EJ Kluhsman BC Graybill MA Liao JZ Schaefer EW . Academic detailing to increase colorectal cancer screening by primary care practices in Appalachian Pennsylvania. BMC Health Serv Res. (2011) 11:112. doi: 10.1186/1472-6963-11-112, PMID: 21600059 PMC3128846

[4] Hossain S Karuniawati H Jairoun AA Urbi Z Ooi DJ John A . Colorectal cancer: A review of carcinogenesis, global epidemiology, current challenges, risk factors, preventive and treatment strategies. Cancers (Basel). (2022) 14:1732. doi: 10.3390/cancers14071732, PMID: 35406504 PMC8996939

[5] Balboa-Barreiro V Pértega-Díaz S García-Rodríguez T González-Martín C Pardeiro-Pértega R Yáñez-González-Dopeso L . Colorectal cancer recurrence and its impact on survival after curative surgery: An analysis based on multistate models. Digestive Liver Dis. (2024) 56:1229–36. doi: 10.1016/j.dld.2023.11.041, PMID: 38087671

[6] Nikolic N Radosavljevic D Gavrilovic D Nikolic V Stanic N Spasic J . Prognostic factors for post-recurrence survival in stage II and III colorectal carcinoma patients. Med (Kaunas). (2021) 57:1108. doi: 10.3390/medicina57101108, PMID: 34684145 PMC8538010

[7] Cañellas-Socias A Cortina C Hernando-Momblona X Palomo-Ponce S Mulholland EJ Turon G . Metastatic recurrence in colorectal cancer arises from residual EMP1+ cells. Nature. (2022) 611:603–13. doi: 10.1038/s41586-022-05402-9, PMID: 36352230 PMC7616986

[8] Caso R Fabrizio A Sosin M . Prolonged follow-up of colorectal cancer patients after 5 years: to follow or not to follow, that is the question (and how)! Ann Transl Med. (2020) 8:164. doi: 10.21037/atm.2019.11.40, PMID: 32309311 PMC7154408

[9] National Comprehensive Cancer Network . NCCN Guidelines Colon Cancer (Version 3.2025) (2023). Available online at: https://www.nccn.org/professionals/physician_gls/pdf/colon.pdf (Accessed January 7, 2026).

[10] National Comprehensive Cancer Network . NCCN Guidelines Rectal Cancer (Version 2.2025) (2025). Available online at: https://www.nccn.org/professionals/physician_gls/pdf/rectal.pdf (Accessed January 7, 2026).

[11] Kumar A Gautam V Sandhu A Rawat K Sharma A Saha L . Current and emerging therapeutic approaches for colorectal cancer: A comprehensive review. World J Gastrointestinal Surg. (2023) 15:495. doi: 10.4240/wjgs.v15.i4.495, PMID: 37206081 PMC10190721

[12] Vantourout P Hayday A . Six-of-the-best: unique contributions of γδ T cells to immunology. Nat Rev Immunol. (2013) 13:88–100. doi: 10.1038/nri3384, PMID: 23348415 PMC3951794

[13] Ribot JC Lopes N Silva-Santos B . γδ T cells in tissue physiology and surveillance. Nat Rev Immunol. (2021) 21:221–32. doi: 10.1038/s41577-020-00452-4, PMID: 33057185

[14] Dong R Zhang Y Xiao H Zeng X . Engineering γδ T cells: recognizing and activating on their own way. Front Immunol. (2022) 13:889051. doi: 10.3389/fimmu.2022.889051, PMID: 35603176 PMC9120431

[15] Edelblum KL Shen L Weber CR Marchiando AM Clay BS Wang Y . Dynamic migration of γδ intraepithelial lymphocytes requires occludin. Proc Natl Acad Sci. (2012) 109:7097–102. doi: 10.1073/pnas.1112519109, PMID: 22511722 PMC3345021

[16] Deusch K Lüling F Reich K Classen M Wagner H Pfeffer K . A major fraction of human intraepithelial lymphocytes simultaneously expresses the gamma/delta T cell receptor, the CD8 accessory molecule and preferentially uses the V delta 1 gene segment. Eur J Immunol. (1991) 21:1053–9. doi: 10.1002/eji.1830210429, PMID: 1826884

[17] Ullrich R Schieferdecker HL Ziegler K Riecken EO Zeitz M . gamma delta T cells in the human intestine express surface markers of activation and are preferentially located in the epithelium. Cell Immunol. (1990) 128:619–27. doi: 10.1016/0008-8749(90)90053-T, PMID: 2113432

[18] Vroom TM Scholte G Ossendorp F Borst J . Tissue distribution of human gamma delta T cells: no evidence for general epithelial tropism. J Clin Pathol. (1991) 44:1012–7. doi: 10.1136/jcp.44.12.1012, PMID: 1838746 PMC494970

[19] Nguyen HT Duong H-Q . The molecular characteristics of colorectal cancer: Implications for diagnosis and therapy (Review). Oncol Lett. (2018) 16:9–18. doi: 10.3892/ol.2018.8679, PMID: 29928381 PMC6006272

[20] Chennupati V Worbs T Liu X Malinarich FH Schmitz S Haas JD . Intra- and intercompartmental movement of γδ T cells: intestinal intraepithelial and peripheral γδ T cells represent exclusive nonoverlapping populations with distinct migration characteristics. J Immunol. (2010) 185:5160–8. doi: 10.4049/jimmunol.1001652, PMID: 20870939

[21] Morikawa R Nemoto Y Yonemoto Y Tanaka S Takei Y Oshima S . Intraepithelial lymphocytes suppress intestinal tumor growth by cell-to-cell contact via CD103/E-cadherin signal. Cell Mol Gastroenterol Hepatol. (2021) 11:1483–503. doi: 10.1016/j.jcmgh.2021.01.014, PMID: 33515805 PMC8025200

[22] Ran R Trapecar M Brubaker DK . Systematic analysis of human colorectal cancer scRNA-seq revealed limited pro-tumoral IL-17 production potential in gamma delta T cells. Neoplasia. (2024) 58:101072. doi: 10.1016/j.neo.2024.101072, PMID: 39454432 PMC11539345

[23] Rancan C Arias-Badia M Dogra P Chen B Aran D Yang H . Exhausted intratumoral Vδ2- γδ T cells in human kidney cancer retain effector function. Nat Immunol. (2023) 24:612–24. doi: 10.1038/s41590-023-01448-7, PMID: 36928415 PMC10063448

[24] Wei F Zhong S Ma Z Kong H Medvec A Ahmed R . Strength of PD-1 signaling differentially affects T-cell effector functions. Proc Natl Acad Sci U.S.A. (2013) 110:E2480–9. doi: 10.1073/pnas.1305394110, PMID: 23610399 PMC3703988

[25] Browaeys R Saelens W Saeys Y . NicheNet: modeling intercellular communication by linking ligands to target genes. Nat Methods. (2020) 17:159–62. doi: 10.1038/s41592-019-0667-5, PMID: 31819264

[26] Theodoris CV Xiao L Chopra A Chaffin MD Al Sayed ZR Hill MC . Transfer learning enables predictions in network biology. Nature. (2023) 618:616–24. doi: 10.1038/s41586-023-06139-9, PMID: 37258680 PMC10949956

[27] Lopes-Ramos CM Paulson JN Chen C-Y Kuijjer ML Fagny M Platig J . Regulatory network changes between cell lines and their tissues of origin. BMC Genomics. (2017) 18:723. doi: 10.1186/s12864-017-4111-x, PMID: 28899340 PMC5596945

[28] Rigau M Ostrouska S Fulford TS Johnson DN Woods K Ruan Z . Butyrophilin 2A1 is essential for phosphoantigen reactivity by γδ T cells. Science. (2020) 367:eaay5516. doi: 10.1126/science.aay5516, PMID: 31919129

[29] Harly C Joyce SP Domblides C Bachelet T Pitard V Mannat C . Human γδ T cell sensing of AMPK-dependent metabolic tumor reprogramming through TCR recognition of EphA2. Sci Immunol. (2021) 6:eaba9010. doi: 10.1126/sciimmunol.aba9010, PMID: 34330813

[30] Willcox CR Pitard V Netzer S Couzi L Salim M Silberzahn T . Cytomegalovirus and tumor stress surveillance by binding of a human γδ T cell antigen receptor to endothelial protein C receptor. Nat Immunol. (2012) 13:872–9. doi: 10.1038/ni.2394, PMID: 22885985

[31] Hwang J-R Byeon Y Kim D Park S-G . Recent insights of T cell receptor-mediated signaling pathways for T cell activation and development. Exp Mol Med. (2020) 52:750–61. doi: 10.1038/s12276-020-0435-8, PMID: 32439954 PMC7272404

[32] Chen Y Lu D Churov A Fu R . Research progress on NK cell receptors and their signaling pathways. Mediators Inflammation. (2020) 2020:1–14. doi: 10.1155/2020/8873152, PMID: 32774149 PMC7396059

[33] Rosales C Uribe-Querol E . Fc receptors: Cell activators of antibody functions. Adv Bioscience Biotechnol. (2013) 4:21–33. doi: 10.4236/abb.2013.44A004

[34] Aibar S González-Blas CB Moerman T Huynh-Thu VA Imrichova H Hulselmans G . SCENIC: single-cell regulatory network inference and clustering. Nat Methods. (2017) 14:1083–6. doi: 10.1038/nmeth.4463, PMID: 28991892 PMC5937676

[35] Oki S Ohta T Shioi G Hatanaka H Ogasawara O Okuda Y . ChIP-Atlas: a data-mining suite powered by full integration of public ChIP-seq data. EMBO Rep. (2018) 19:e46255. doi: 10.15252/embr.201846255, PMID: 30413482 PMC6280645

[36] Zou Z Ohta T Oki S . ChIP-Atlas 3.0: a data-mining suite to explore chromosome architecture together with large-scale regulome data. Nucleic Acids Res. (2024) 52:W45–53. doi: 10.1093/nar/gkae358, PMID: 38749504 PMC11223792

[37] Zou Z Ohta T Miura F Oki S . ChIP-Atlas 2021 update: a data-mining suite for exploring epigenomic landscapes by fully integrating ChIP-seq, ATAC-seq and Bisulfite-seq data. Nucleic Acids Res. (2022) 50:W175–82. doi: 10.1093/nar/gkac199, PMID: 35325188 PMC9252733

[38] Kamimoto K Stringa B Hoffmann CM Jindal K Solnica-Krezel L Morris SA . Dissecting cell identity via network inference and in silico gene perturbation. Nature. (2023) 614:742–51. doi: 10.1038/s41586-022-05688-9, PMID: 36755098 PMC9946838

[39] Joanito I Wirapati P Zhao N Nawaz Z Yeo G Lee F . Single-cell and bulk transcriptome sequencing identifies two epithelial tumor cell states and refines the consensus molecular classification of colorectal cancer. Nat Genet. (2022) 54:963–75. doi: 10.1038/s41588-022-01100-4, PMID: 35773407 PMC9279158

[40] Musa J Aynaud M-M Mirabeau O Delattre O Grünewald TG . MYBL2 (B-Myb): a central regulator of cell proliferation, cell survival and differentiation involved in tumorigenesis. Cell Death Dis. (2017) 8:e2895–5. doi: 10.1038/cddis.2017.244, PMID: 28640249 PMC5520903

[41] Li Q Zhang L You W Xu J Dai J Hua D . PRDM1/BLIMP1 induces cancer immune evasion by modulating the USP22-SPI1-PD-L1 axis in hepatocellular carcinoma cells. Nat Commun. (2022) 13:7677. doi: 10.1038/s41467-022-35469-x, PMID: 36509766 PMC9744896

[42] Doan AE Mueller KP Chen AY Rouin GT Chen Y Daniel B . FOXO1 is a master regulator of memory programming in CAR T cells. Nature. (2024) 629:211–8. doi: 10.1038/s41586-024-07300-8, PMID: 38600391 PMC11062920

[43] Hsu P Choi EJ Wong WH Lin YH Vandenburgh SA Liu YC . Foxo1 regulates intestinal tissue–resident memory CD8 T cell biology in an anatomic compartment– and context-specific manner. Sci Immunol. (2025) 10:eadn1894. doi: 10.1126/sciimmunol.adn1894, PMID: 40215325 PMC12955362

[44] Carr TM Wheaton JD Houtz GM Ciofani M . JunB promotes Th17 cell identity and restrains alternative CD4+ T-cell programs during inflammation. Nat Commun. (2017) 8:301. doi: 10.1038/s41467-017-00380-3, PMID: 28824171 PMC5563507

[45] Lyon de Ana C Arakcheeva K Agnihotri P Derosia N Winandy S . Lack of Ikaros deregulates inflammatory gene programs in T cells. J Immunol. (2019) 202:1112–23. doi: 10.4049/jimmunol.1801270, PMID: 30635395 PMC6365004

[46] Stelekati E Chen Z Manne S Kurachi M Ali M-A Lewy K . Long-term persistence of exhausted CD8 T cells in chronic infection is regulated by microRNA-155. Cell Rep. (2018) 23:2142–56. doi: 10.1016/j.celrep.2018.04.038, PMID: 29768211 PMC5986283

[47] Skon CN Lee J-Y Anderson KG Masopust D Hogquist KA Jameson SC . Transcriptional downregulation of S1pr1 is required for establishment of resident memory CD8+ T cells. Nat Immunol. (2013) 14:1285–93. doi: 10.1038/ni.2745, PMID: 24162775 PMC3844557

[48] Bai A Hu H Yeung M Chen J . Krüppel-like factor 2 controls T cell trafficking by activating L-selectin (CD62L) and sphingosine-1-phosphate receptor 1 transcription1. J Immunol. (2007) 178:7632–9. doi: 10.4049/jimmunol.178.12.7632, PMID: 17548599

[49] Matloubian M Lo CG Cinamon G Lesneski MJ Xu Y Brinkmann V . Lymphocyte egress from thymus and peripheral lymphoid organs is dependent on S1P receptor 1. Nature. (2004) 427:355–60. doi: 10.1038/nature02284, PMID: 14737169

[50] Takada K Wang X Hart GT Odumade OA Weinreich MA Hogquist KA . Kruppel-like factor 2 is required for trafficking but not quiescence in postactivated T cells. J Immunol. (2011) 186:775–83. doi: 10.4049/jimmunol.1000094, PMID: 21160050 PMC3017213

[51] Fagerberg E Attanasio J Dien C Singh J Kessler EA Abdullah L . KLF2 maintains lineage fidelity and suppresses CD8 T cell exhaustion during acute LCMV infection. Science. (2025) 387:eadn2337. doi: 10.1126/science.adn2337, PMID: 39946463 PMC12199163

[52] Brien JD Daffis S Lazear HM Cho H Suthar MS Jr MG . Interferon regulatory factor-1 (IRF-1) shapes both innate and CD8+ T cell immune responses against west nile virus infection. PLOS Pathogens. (2011) 7:e1002230. doi: 10.1371/journal.ppat.1002230, PMID: 21909274 PMC3164650

[53] Uyeda MJ Freeborn RA Cieniewicz B Romano R Chen P Liu JM-H . BHLHE40 regulates IL-10 and IFN-γ Production in T cells but does not interfere with human type 1 regulatory T cell differentiation. Frontiers in Immunology. (2021) 12:683680. doi: 10.3389/fimmu.2021.683680, PMID: 34305917 PMC8293608

[54] Borys SM Bag AK Brossay L Adeegbe DO . The yin and yang of targeting KLRG1+ Tregs and effector cells. Front Immunol. (2022) 13. doi: 10.3389/fimmu.2022.894508, PMID: 35572605 PMC9098823

[55] Melis L Praet L Pircher H Venken K Elewaut D . Senescence marker killer cell lectin-like receptor G1 (KLRG1) contributes to TNF- production by interaction with its soluble E-cadherin ligand in chronically inflamed joints. Ann rheumatic Dis. (2013) 73:1223–31. doi: 10.1136/annrheumdis-2013-203881, PMID: 23740233

[56] Zhang Y Chen S Tang X Peng Y Jiang T Zhang X . The role of KLRG1: a novel biomarker and new therapeutic target. Cell Communication Signaling. (2024) 22:337. doi: 10.1186/s12964-024-01714-7, PMID: 38898461 PMC11186184

[57] Tata A Dodard G Fugère C Leget C Ors M Rossi B . Combination blockade of KLRG1 and PD-1 promotes immune control of local and disseminated cancers. Oncoimmunology. (2021) 10:1933808. doi: 10.1080/2162402X.2021.1933808, PMID: 34188973 PMC8208121

[58] Li L Wan S Tao K Wang G Zhao E . KLRG1 restricts memory T cell antitumor immunity. Oncotarget. (2016) 7:61670–8. doi: 10.18632/oncotarget.11430, PMID: 27557510 PMC5308681

[59] Wang JM Cheng YQ Shi L Ying RS Wu XY Li GY . KLRG1 negatively regulates natural killer cell functions through the akt pathway in individuals with chronic hepatitis C virus infection. J Virol. (2013) 87:11626–36. doi: 10.1128/JVI.01515-13, PMID: 23966413 PMC3807337

[60] Zakeri N Hall A Swadling L Pallett LJ Schmidt NM Diniz MO . Characterisation and induction of tissue-resident gamma delta T-cells to target hepatocellular carcinoma. Nat Commun. (2022) 13:1372. doi: 10.1038/s41467-022-29012-1, PMID: 35296658 PMC8927126

[61] Herndler-Brandstetter D Ishigame H Shinnakasu R Plajer V Stecher C Zhao J . KLRG1+ Effector CD8+ T cells lose KLRG1, differentiate into all memory T cell lineages, and convey enhanced protective immunity. Immunity. (2018) 48:716–729.e8. doi: 10.1016/j.immuni.2018.03.015, PMID: 29625895 PMC6465538

[62] Vermijlen D Brouwer M Donner C Liesnard C Tackoen M Van Rysselberge M . Human cytomegalovirus elicits fetal γδ T cell responses. Utero J Exp Med. (2010) 207:807–21. doi: 10.1084/jem.20090348, PMID: 20368575 PMC2856038

[63] Gibbons DL Haque SFY Silberzahn T Hamilton K Langford C Ellis P . Neonates harbour highly active γδ T cells with selective impairments in preterm infants. Eur J Immunol. (2009) 39:1794–806. doi: 10.1002/eji.200939222, PMID: 19544311

[64] Yared N Papadopoulou M Barennes P Pham H-P Quiniou V Netzer S . Long-lived central memory γδ T cells confer protection against murine cytomegalovirus reinfection. PloS Pathog. (2024) 20:e1010785. doi: 10.1371/journal.ppat.1010785, PMID: 38976755 PMC11257398

[65] Masuda K Kornberg A Miller J Lin S Suek N Botella T . Multiplexed single-cell analysis reveals prognostic and nonprognostic T cell types in human colorectal cancer. JCI Insight. (2022) 7:e154646. doi: 10.1172/jci.insight.154646, PMID: 35192548 PMC9057629

[66] Vieira Braga FA Hertoghs KML Kragten NAM Doody GM Barnes NA Remmerswaal EBM . Blimp-1 homolog Hobit identifies effector-type lymphocytes in humans. Eur J Immunol. (2015) 45:2945–58. doi: 10.1002/eji.201545650, PMID: 26179882

[67] Parga-Vidal L Behr FM Kragten NAM Nota B Wesselink TH Kavazović I . Hobit identifies tissue-resident memory T cell precursors that are regulated by Eomes. Sci Immunol. (2021) 6:eabg3533. doi: 10.1126/sciimmunol.abg3533, PMID: 34417257

[68] Nolz JC Richer MJ . Control of memory CD8+ T cell longevity and effector functions by IL-15. Mol Immunol. (2020) 117:180–8. doi: 10.1016/j.molimm.2019.11.011, PMID: 31816491 PMC6928418

[69] Aehnlich P Carnaz Simões AM Skadborg SK Holmen Olofsson G Thor Straten P . Expansion with IL-15 increases cytotoxicity of Vγ9Vδ2 T cells and is associated with higher levels of cytotoxic molecules and T-bet. Front Immunol. (2020) 11:1868. doi: 10.3389/fimmu.2020.01868, PMID: 32983105 PMC7485111

[70] Wang H Wang X Wang W Chai W Song W Zhang H . Interleukin-15 enhanced the survival of human γδT cells by regulating the expression of Mcl-1 in neuroblastoma. Cell Death Discov. (2022) 8:1–11. doi: 10.1038/s41420-022-00942-5, PMID: 35351861 PMC8964681

[71] Ferry GM Agbuduwe C Forrester M Dunlop S Chester K Fisher J . A simple and robust single-step method for CAR-Vδ1 γδT cell expansion and transduction for cancer immunotherapy. Front Immunol. (2022) 13. doi: 10.3389/fimmu.2022.863155, PMID: 35711450 PMC9197253

[72] Felices M Lenvik A Chu S McElmurry R Cooley S Tolar J . Continuous IL-15 signaling leads to functional exhaustion of human natural killer cells through metabolic changes that alters their. In Vivo Anti-Tumor Activity Blood. (2016) 128:551. doi: 10.1182/blood.V128.22.551.551

[73] Felices M Lenvik AJ McElmurry R Chu S Hinderlie P Bendzick L . Continuous treatment with IL-15 exhausts human NK cells via a metabolic defect. JCI Insight. (2018). 3:e96219. doi: 10.1172/jci.insight.96219, PMID: 29415897 PMC5821201

[74] Tian Y Zajac AJ . IL-21 and T cell differentiation: consider the context. Trends Immunol. (2016) 37:557–68. doi: 10.1016/j.it.2016.06.001, PMID: 27389961 PMC4969098

[75] Vermijlen D Ellis P Langford C Klein A Engel R Willimann K . Distinct cytokine-driven responses of activated blood γδ T cells: insights into unconventional T cell pleiotropy. J Immunol. (2007) 178:4304–14. doi: 10.4049/jimmunol.178.7.4304, PMID: 17371987 PMC3915340

[76] Thedrez A Harly C Morice A Salot S Bonneville M Scote E . IL-21-mediated potentiation of antitumor cytolytic and proinflammatory responses of human Vγ9Vδ2 T cells for adoptive immunotherapy1. J Immunol. (2009) 182:3423–31. doi: 10.4049/jimmunol.0803068, PMID: 19265120

[77] Zeng R Spolski R Finkelstein SE Oh S Kovanen PE Hinrichs CS . Synergy of IL-21 and IL-15 in regulating CD8+ T cell expansion and function. J Exp Med. (2005) 201:139–48. doi: 10.1084/jem.20041057, PMID: 15630141 PMC2212766

[78] Zhang C Kadu S Xiao Y Johnson O Kelly A O’Connor RS . Sequential exposure to IL21 and IL15 during human natural killer cell expansion optimizes yield and function. Cancer Immunol Res. (2023) 11:1524–37. doi: 10.1158/2326-6066.CIR-23-0151, PMID: 37649085 PMC10618651

[79] Bullock TNJ . CD40 stimulation as a molecular adjuvant for cancer vaccines and other immunotherapies. Cell Mol Immunol. (2022) 19:14–22. doi: 10.1038/s41423-021-00734-4, PMID: 34282297 PMC8752810

[80] Tay NQ Lee DCP Chua YL Prabhu N Gascoigne NRJ Kemeny DM . CD40L expression allows CD8+ T cells to promote their own expansion and differentiation through dendritic cells. Front Immunol. (2017) 8. doi: 10.3389/fimmu.2017.01484, PMID: 29163545 PMC5672143

[81] Wu R Ohara RA Jo S Liu T-T Ferris ST Ou F . Mechanisms of CD40-dependent cDC1 licensing beyond costimulation. Nat Immunol. (2022) 23:1536–50. doi: 10.1038/s41590-022-01324-w, PMID: 36271147 PMC9896965

[82] Inoue S-I Niikura M Takeo S Mineo S Kawakami Y Uchida A . Enhancement of dendritic cell activation via CD40 ligand-expressing γδ T cells is responsible for protective immunity to Plasmodium parasites. Proc Natl Acad Sci. (2012) 109:12129–34. doi: 10.1073/pnas.1204480109, PMID: 22778420 PMC3409789

[83] Bourgeois C Rocha B . & Tanchot, C. A role for CD40 expression on CD8+ T cells in the generation of CD8+ T cell memory. Science. (2002) 297:2060–3. doi: 10.1126/science.1072615, PMID: 12242444

[84] Choi H Lee H-J Sohn H-J Kim T-G . CD40 ligand stimulation affects the number and memory phenotypes of human peripheral CD8+ T cells. BMC Immunol. (2023) 24:15. doi: 10.1186/s12865-023-00547-2, PMID: 37391734 PMC10311846

[85] Singh R Kim Y-H Lee S-J Eom H-S Choi BK . 4-1BB immunotherapy: advances and hurdles. Exp Mol Med. (2024) 56:32–9. doi: 10.1038/s12276-023-01136-4, PMID: 38172595 PMC10834507

[86] Cappell KM . & Kochenderfer, J. N. A comparison of chimeric antigen receptors containing CD28 versus 4-1BB costimulatory domains. Nat Rev Clin Oncol. (2021) 18:715–27. doi: 10.1038/s41571-021-00530-z, PMID: 34230645

[87] Acuto O Michel F . CD28-mediated co-stimulation: a quantitative support for TCR signalling. Nat Rev Immunol. (2003) 3:939–51. doi: 10.1038/nri1248, PMID: 14647476

[88] Cordova A Toia F Mendola CL Orlando V Meraviglia S Rinaldi G . Characterization of human γδ T lymphocytes infiltrating primary Malignant melanomas. PloS One. (2012) 7:e49878. doi: 10.1371/journal.pone.0049878, PMID: 23189169 PMC3506540

[89] Gray JI Caron DP Wells SB Guyer R Szabo P Rainbow D . Human γδ T cells in diverse tissues exhibit site-specific maturation dynamics across the life span. Sci Immunol. (2024) 9:eadn3954. doi: 10.1126/sciimmunol.adn3954, PMID: 38848342 PMC11425769

[90] Pizzolato G Kaminski H Tosolini M Franchini D-M Pont F Martins F . Single-cell RNA sequencing unveils the shared and the distinct cytotoxic hallmarks of human TCRVδ1 and TCRVδ2 γδ T lymphocytes. Proc Natl Acad Sci. (2019) 116:11906–15. doi: 10.1073/pnas.1818488116, PMID: 31118283 PMC6576116

[91] Stary V Pandey RV List J Kleissl L Deckert F Kabiljo J . Dysfunctional tumor-infiltrating Vδ1 + T lymphocytes in microsatellite-stable colorectal cancer. Nat Commun. (2024) 15:6949. doi: 10.1038/s41467-024-51025-1, PMID: 39138181 PMC11322529

[92] Rodin W Szeponik L Rangelova T Tamiru Kebede F Österlund T Sundström P . γδ T cells in human colon adenocarcinomas comprise mainly Vδ1, Vδ2, and Vδ3 cells with distinct phenotype and function. Cancer Immunol Immunother. (2024) 73:174. doi: 10.1007/s00262-024-03758-7, PMID: 38953978 PMC11219682

[93] Ran R Uslu M Siddiqui MF Brubaker DK Trapecar M . Single-cell analysis reveals tissue-specific T cell adaptation and clonal distribution across the human gut-liver-blood axis. (2025). doi: 10.1101/2025.03.11.642626, PMID: 40161783 PMC11952442

[94] Ohteki T MacDonald HR . Expression of the CD28 costimulatory molecule on subsets of murine intestinal intraepithelial lymphocytes correlates with lineage and responsiveness. Eur J Immunol. (1993) 23:1251–5. doi: 10.1002/eji.1830230609, PMID: 8099014

[95] De Rosa SC Mitra DK Watanabe N Herzenberg LA Herzenberg LA Roederer M . Vδ1 and Vδ2 γδ T cells express distinct surface markers and might be developmentally distinct lineages. J Leukocyte Biol. (2001) 70:518–26. doi: 10.1189/jlb.70.4.518, PMID: 11590187

[96] Testi R Lanier LL . Functional expression of CD28 on T cell antigen receptor γ/δ-bearing T lymphocytes. Eur J Immunol. (1989) 19:185–8. doi: 10.1002/eji.1830190129, PMID: 2537735

[97] Ribot JC deBarros A Mancio-Silva L Pamplona A Silva-Santos B . B7–CD28 costimulatory signals control the survival and proliferation of murine and human γδ T cells via IL-2 production. J Immunol. (2012) 189:1202–8. doi: 10.4049/jimmunol.1200268, PMID: 22732586

[98] Pei Y Wen K Xiang Z Huang C Wang X Mu X . CD137 costimulation enhances the antiviral activity of Vγ9Vδ2-T cells against influenza virus. Sig Transduct Target Ther. (2020) 5:1–10. doi: 10.1038/s41392-020-0174-2, PMID: 32488072 PMC7266814

[99] deBarros A Chaves-Ferreira M d’Orey F Ribot JC Silva-Santos B . CD70–CD27 interactions provide survival and proliferative signals that regulate T cell receptor-driven activation of human γδ peripheral blood lymphocytes. Eur J Immunol. (2011) 41:195–201. doi: 10.1002/eji.201040905, PMID: 21182090

[100] Kato Y Tanaka Y Hayashi M Okawa K Minato N . Involvement of CD166 in the activation of human γδT cells by tumor cells sensitized with nonpeptide antigens1. J Immunol. (2006) 177:877–84. doi: 10.4049/jimmunol.177.2.877, PMID: 16818742

[101] Von Lilienfeld-Toal M Nattermann J Feldmann G Sievers E Frank S Strehl J . Activated γδ T cells express the natural cytotoxicity receptor natural killer p44 and show cytotoxic activity against myeloma cells. Clin Exp Immunol. (2006) 144:528–33. doi: 10.1111/j.1365-2249.2006.03078.x, PMID: 16734623 PMC1941970

[102] Correia DV Fogli M Hudspeth K da Silva MG Mavilio D Silva-Santos B . Differentiation of human peripheral blood Vδ1+ T cells expressing the natural cytotoxicity receptor NKp30 for recognition of lymphoid leukemia cells. Blood. (2011) 118:992–1001. doi: 10.1182/blood-2011-02-339135, PMID: 21633088

[103] Silva-Santos B Strid J . Working in “NK mode”: natural killer group 2 member D and natural cytotoxicity receptors in stress-surveillance by γδ T cells. Front Immunol. (2018) 9. doi: 10.3389/fimmu.2018.00851, PMID: 29740448 PMC5928212

[104] Paul S Lal G . The molecular mechanism of natural killer cells function and its importance in cancer immunotherapy. Frontiers in Immunology Sec. Cancer Immunity and Immunotherapy. (2017) 8. doi: 10.3389/fimmu.2017.01124, PMID: 28955340 PMC5601256

[105] Lanier LL Corliss B Wu J Phillips JH . Association of DAP12 with activating CD94/NKG2C NK cell receptors. Immunity. (1998) 8:693–701. doi: 10.1016/S1074-7613(00)80574-9, PMID: 9655483

[106] Heumos L Schaar AC Lance C Litinetskaya A Drost F Zappia L . Best practices for single-cell analysis across modalities. Nat Rev Genet. (2023) 24:550–72. doi: 10.1038/s41576-023-00586-w, PMID: 37002403 PMC10066026

[107] Cheng J Zhang J Wu Z Sun X . Inferring microenvironmental regulation of gene expression from single-cell RNA sequencing data using scMLnet with an application to COVID-19. Brief Bioinform. (2021) 22:988–1005. doi: 10.1093/bib/bbaa327, PMID: 33341869 PMC7799217

[108] Baruzzo G Cesaro G Di Camillo B . Identify, quantify and characterize cellular communication from single-cell RNA sequencing data with scSeqComm. Bioinformatics. (2022) 38:1920–9. doi: 10.1093/bioinformatics/btac036, PMID: 35043939

[109] Biancalani T Scalia G Buffoni L Avasthi R Lu Z Sanger A . Deep learning and alignment of spatially resolved single-cell transcriptomes with Tangram. Nat Methods. (2021) 18:1352–62. doi: 10.1038/s41592-021-01264-7, PMID: 34711971 PMC8566243

[110] Foote MB Argilés G Rousseau B Segal NH . Facts and hopes in colorectal cancer immunotherapy. Clin Cancer Res. (2023) 29:4032–9. doi: 10.1158/1078-0432.CCR-22-2176, PMID: 37326624

[111] Larson RC Maus MV . Recent advances and discoveries in the mechanisms and functions of CAR T cells. Nat Rev Cancer. (2021) 21:145–61. doi: 10.1038/s41568-020-00323-z, PMID: 33483715 PMC8353572

[112] Herpers B Eppink B James MI Cortina C Cañellas-Socias A Boj SF . Functional patient-derived organoid screenings identify MCLA-158 as a therapeutic EGFR × LGR5 bispecific antibody with efficacy in epithelial tumors. Nat Cancer. (2022) 3:418–36. doi: 10.1038/s43018-022-00359-0, PMID: 35469014

[113] Pelka K Hofree M Chen JH Sarkizova S Pirl JD Jorgji V . Spatially organized multicellular immune hubs in human colorectal cancer. Cell. (2021) 184:4734–4752.e20. doi: 10.1016/j.cell.2021.08.003, PMID: 34450029 PMC8772395

[114] Khaliq AM Erdogan C Kurt Z Turgut SS Grunvald MW Rand T . Refining colorectal cancer classification and clinical stratification through a single-cell atlas. Genome Biol. (2022) 23:113. doi: 10.1186/s13059-022-02677-z, PMID: 35538548 PMC9092724

[115] Liu X Wang X Yang Q Luo L Liu Z Ren X . Th17 cells secrete TWEAK to trigger epithelial-mesenchymal transition and promote colorectal cancer liver metastasis. Cancer Res. (2024) 84:1352–71. doi: 10.1158/0008-5472.CAN-23-2123, PMID: 38335276

[116] Hsu W-H LaBella KA Lin Y Xu P Lee R Hsieh C-E . Oncogenic KRAS drives lipofibrogenesis to promote angiogenesis and colon cancer progression. Cancer Discov. (2023) 13:2652–73. doi: 10.1158/2159-8290.CD-22-1467, PMID: 37768068 PMC10807546

[117] Guo W Zhang C Wang X Dou D Chen D Li J . Resolving the difference between left-sided and right-sided colorectal cancer by single-cell sequencing. JCI Insight. (2022) 7:e152616. doi: 10.1172/jci.insight.152616, PMID: 34793335 PMC8765049

[118] Zheng X Song J Yu C Zhou Z Liu X Yu J . Single-cell transcriptomic profiling unravels the adenoma-initiation role of protein tyrosine kinases during colorectal tumorigenesis. Signal Transduct Target Ther. (2022) 7:60. doi: 10.1038/s41392-022-00881-8, PMID: 35221332 PMC8882672

[119] Zhang L Yu X Zheng L Zhang Y Li Y Fang Q . Lineage tracking reveals dynamic relationships of T cells in colorectal cancer. Nature. (2018) 564:268–72. doi: 10.1038/s41586-018-0694-x, PMID: 30479382

[120] Che L-H Liu J-W Huo J-P Luo R Xu R-M He C . A single-cell atlas of liver metastases of colorectal cancer reveals reprogramming of the tumor microenvironment in response to preoperative chemotherapy. Cell Discov. (2021) 7:80. doi: 10.1038/s41421-021-00312-y, PMID: 34489408 PMC8421363

[121] Stuart T Butler A Hoffman P Hafemeister C Papalexi E Mauck WM . Comprehensive integration of single-cell data. Cell. (2019) 177:1888–1902.e21. doi: 10.1016/j.cell.2019.05.031, PMID: 31178118 PMC6687398

[122] Wolf FA Angerer P Theis FJ . SCANPY: large-scale single-cell gene expression data analysis. Genome Biol. (2018) 19:15. doi: 10.1186/s13059-017-1382-0, PMID: 29409532 PMC5802054

[123] Welcome to CellOracle’s documentation! — celloracle 0.18.0 documentation. Available online at: https://morris-lab.github.io/CellOracle.documentation/ (Accessed January 7, 2026).

