# Supplementary figures and images for: A ligand-centered framework for γδ T cell activation in colorectal cancer revealed by single-cell and transformer-based perturbation
Source: Front Immunol. 2026 Jan 13;16:1715827. doi: 10.3389/fimmu.2025.1715827 (PMC12835328; doi:10.3389/fimmu.2025.1715827)

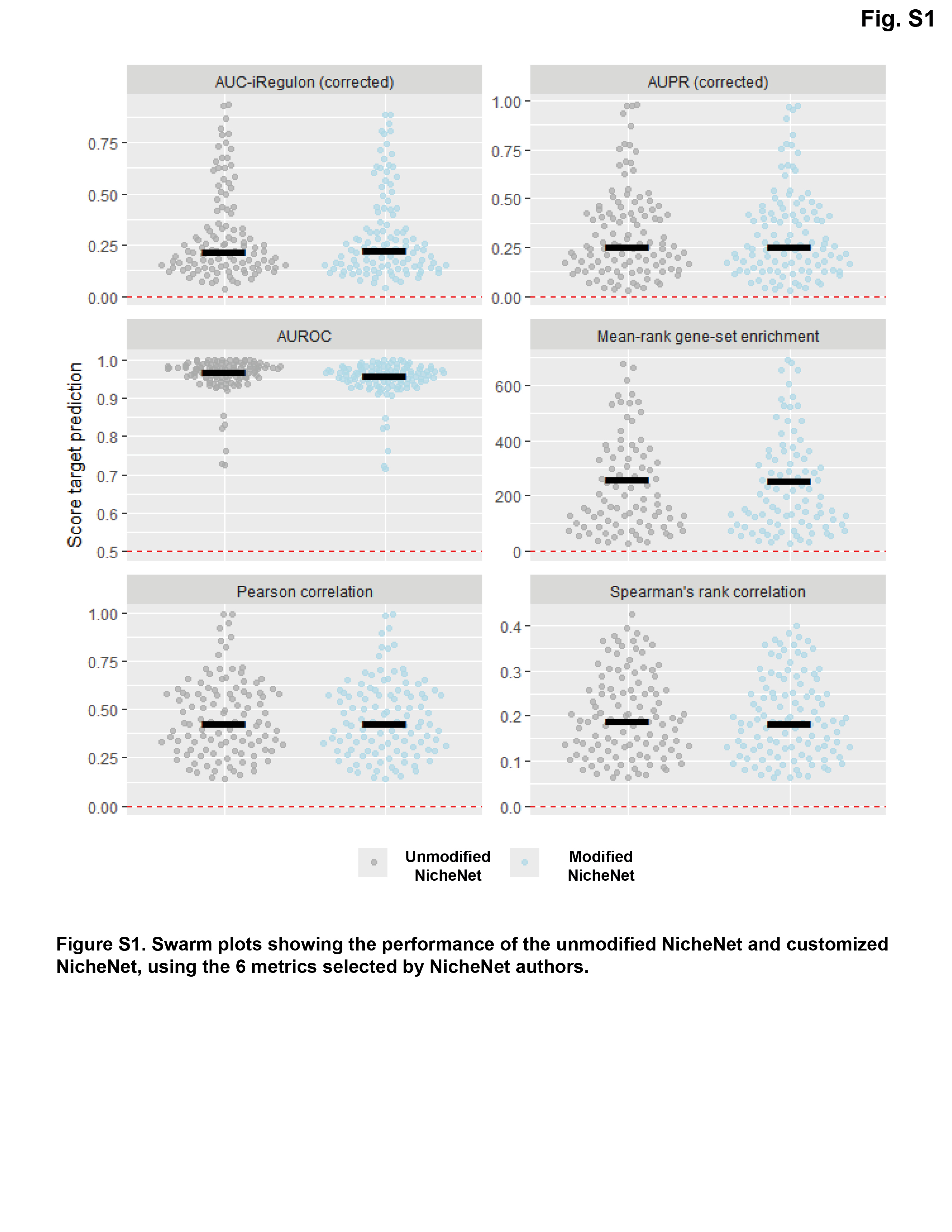

Supplement: Supplementary file 1 [file Image1.tif]

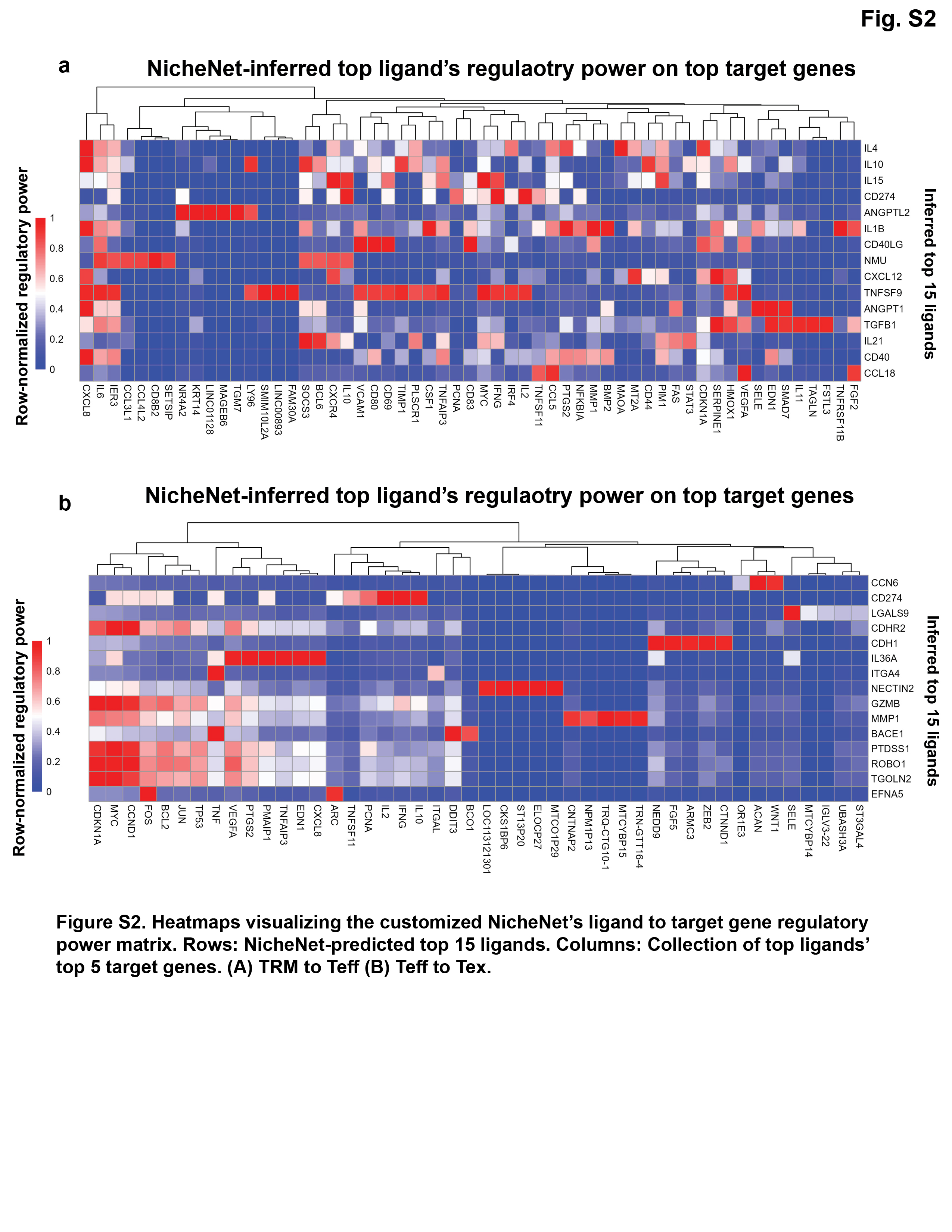

Supplement: Supplementary file 2 [file Image2.tif]

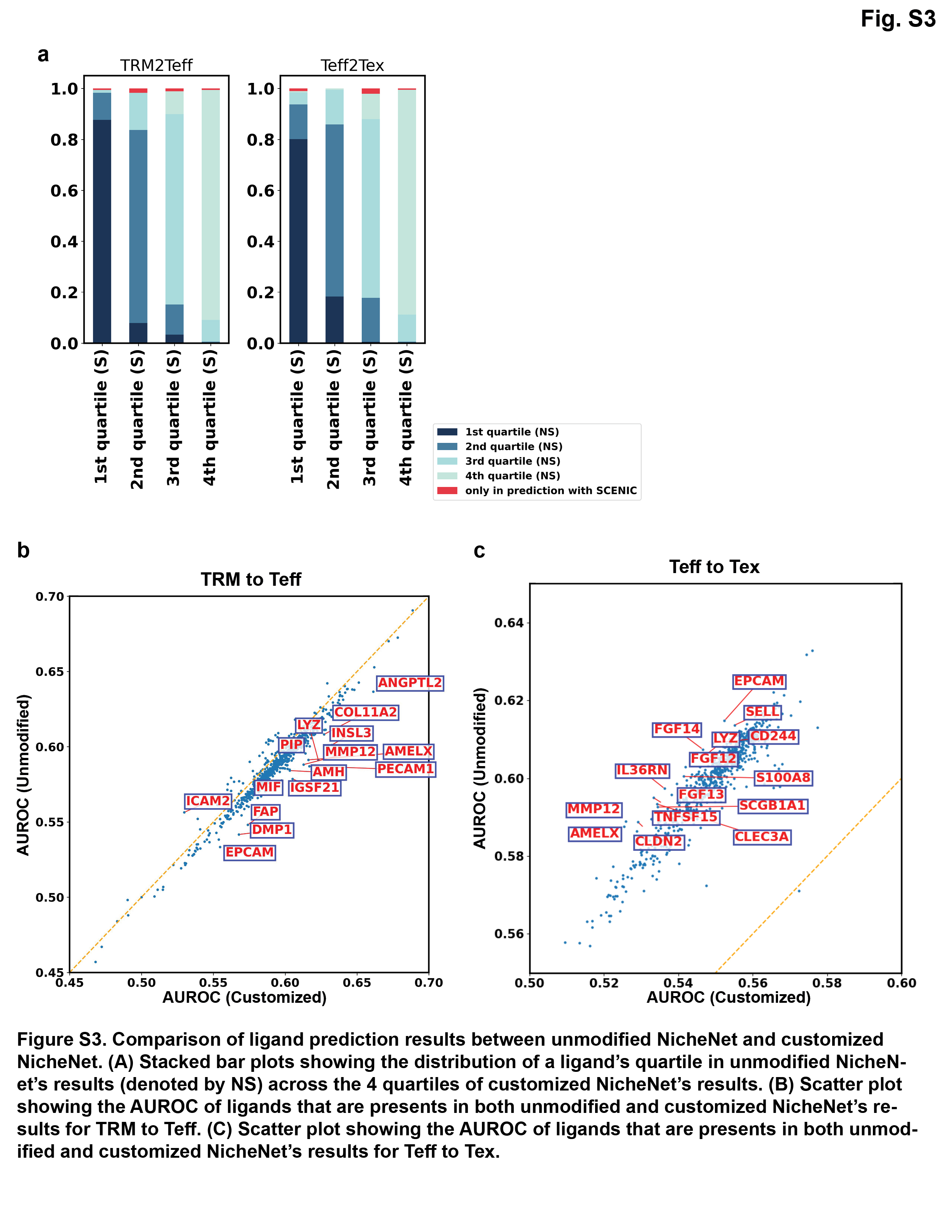

Supplement: Supplementary file 3 [file Image3.tif]

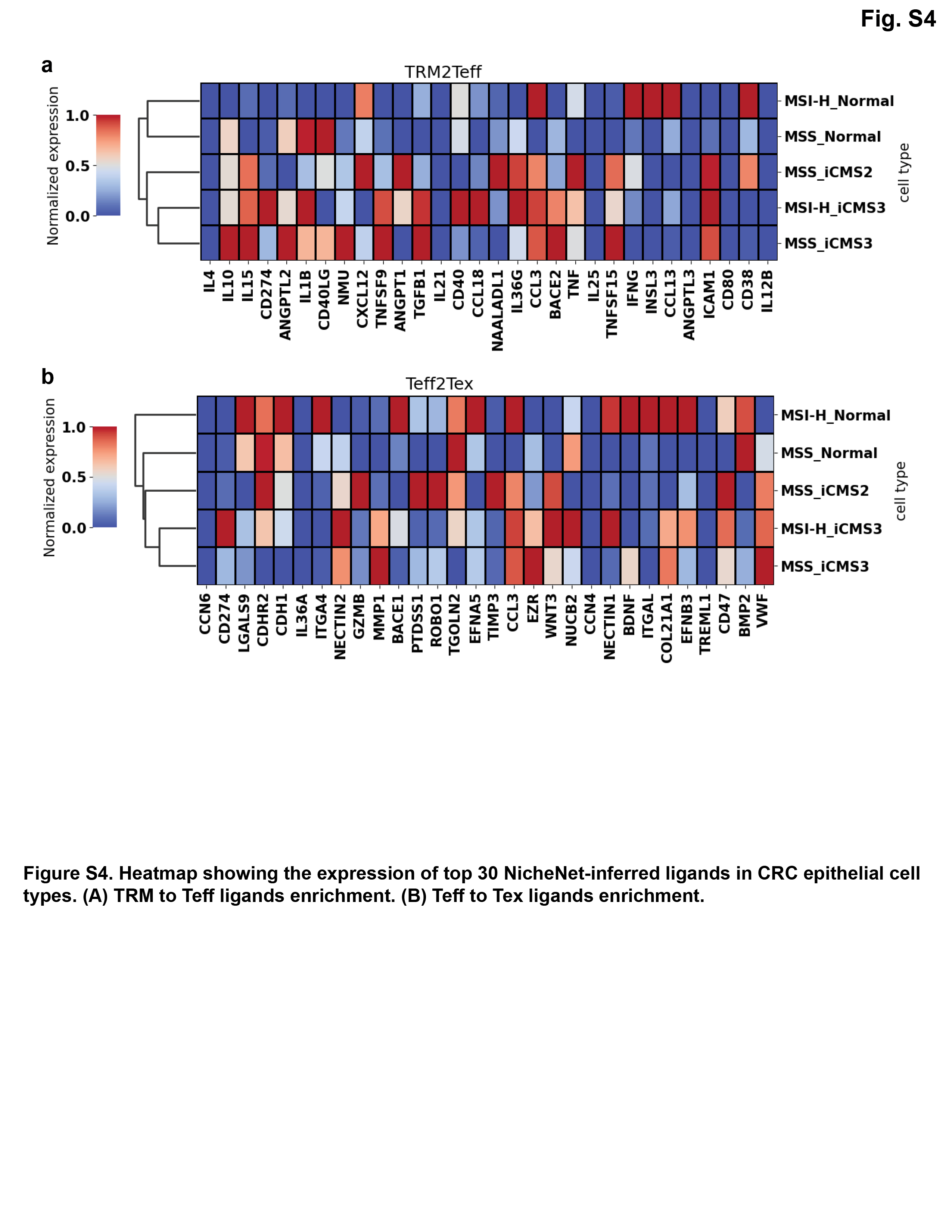

Supplement: Supplementary file 4 [file Image4.tif]

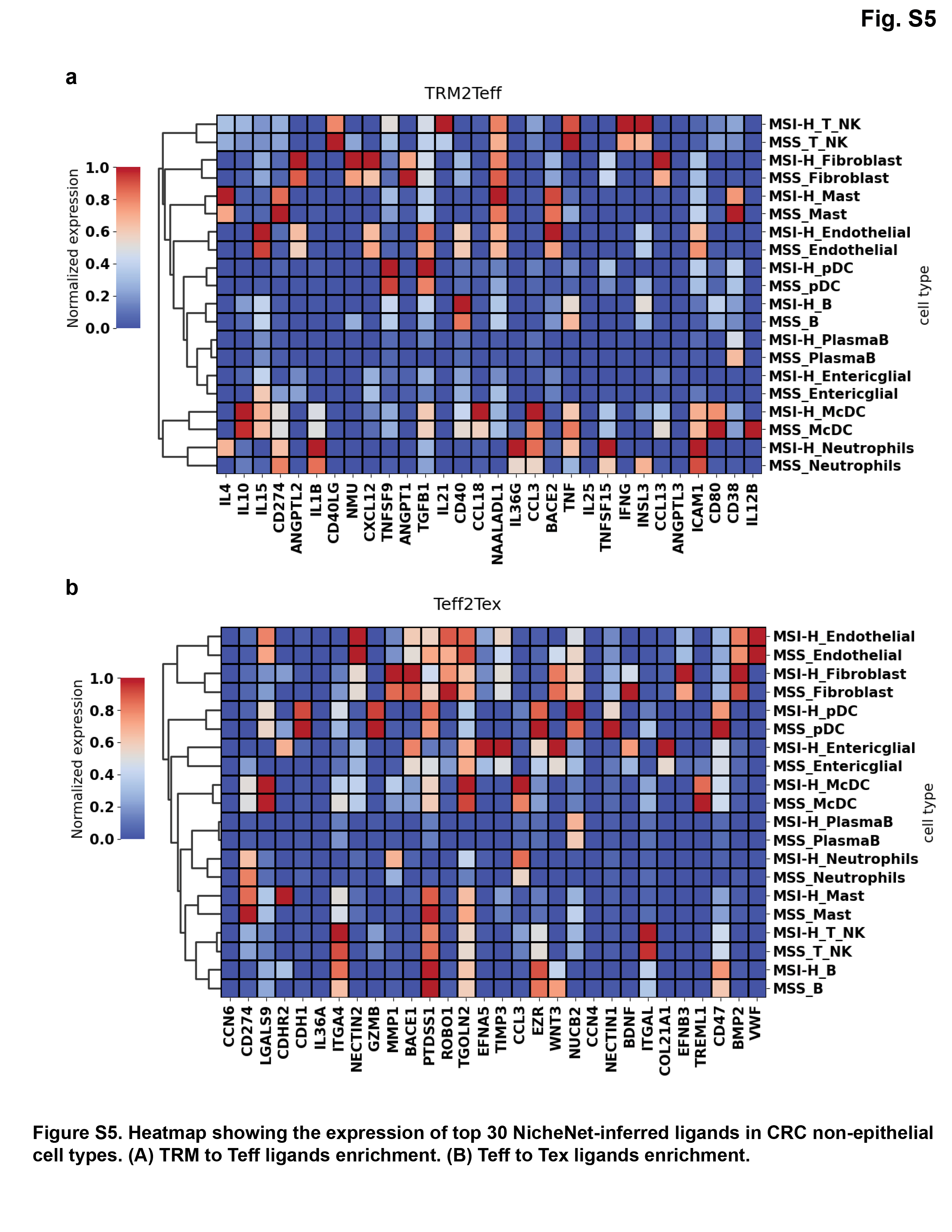

Supplement: Supplementary file 5 [file Image5.tif]

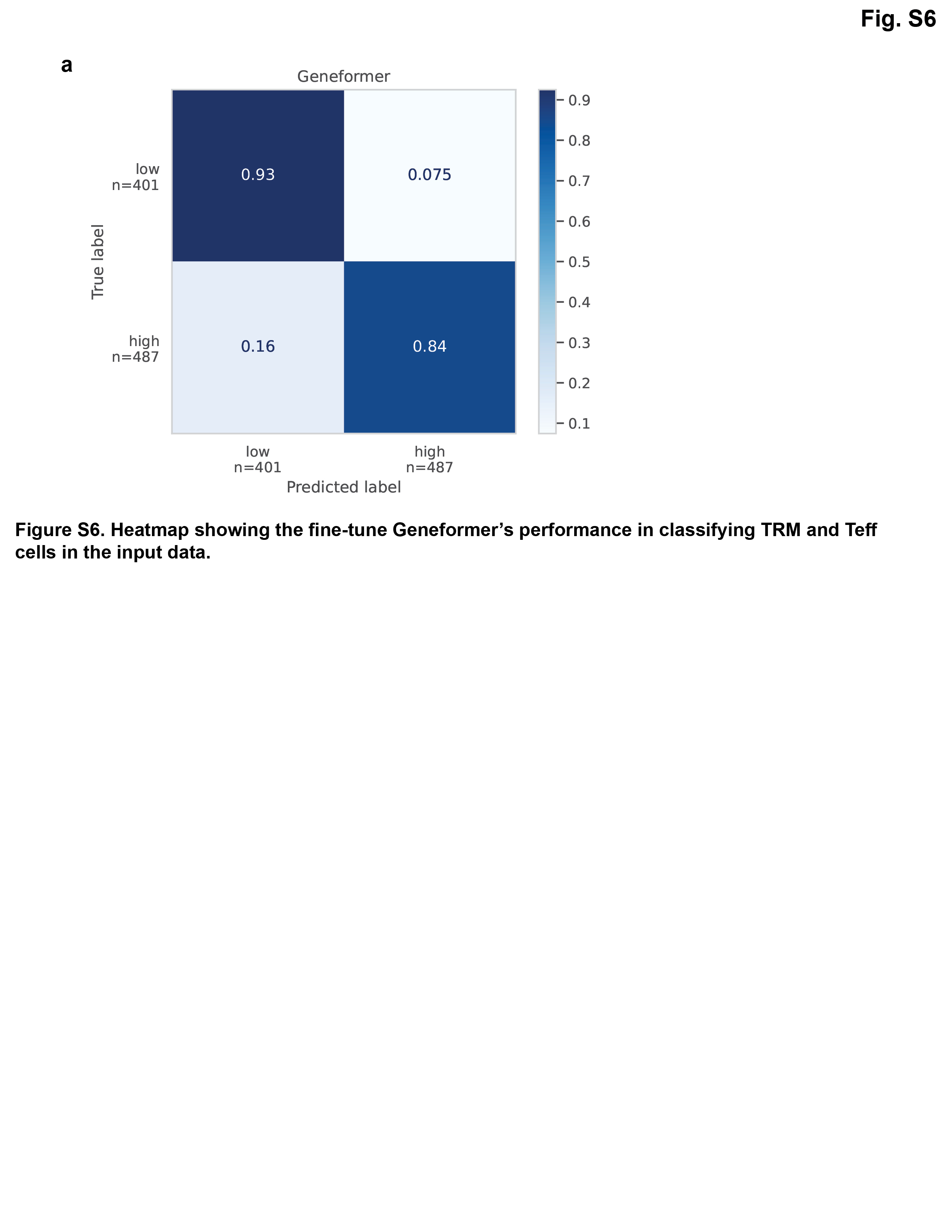

Supplement: Supplementary file 6 [file Image6.tif]
